# Natural Gums in Drug-Loaded Micro- and Nanogels

**DOI:** 10.3390/pharmaceutics15030759

**Published:** 2023-02-24

**Authors:** Anna Froelich, Emilia Jakubowska, Barbara Jadach, Piotr Gadziński, Tomasz Osmałek

**Affiliations:** Chair and Department of Pharmaceutical Technology, Poznan University of Medical Sciences, Grunwaldzka 6, 60-780 Poznan, Poland

**Keywords:** arabic gum, xanthan gum, gellan gum, guar gum, alginate, polysaccharide, drug delivery, microgel, nanogel

## Abstract

Gums are polysaccharide compounds obtained from natural sources, such as plants, algae and bacteria. Because of their excellent biocompatibility and biodegradability, as well as their ability to swell and their sensitivity to degradation by the colon microbiome, they are regarded as interesting potential drug carriers. In order to obtain properties differing from the original compounds, blends with other polymers and chemical modifications are usually applied. Gums and gum-derived compounds can be applied in the form of macroscopic hydrogels or can be formulated into particulate systems that can deliver the drugs via different administration routes. In this review, we present and summarize the most recent studies regarding micro- and nanoparticles obtained with the use of gums extensively investigated in pharmaceutical technology, their derivatives and blends with other polymers. This review focuses on the most important aspects of micro- and nanoparticulate systems formulation and their application as drug carriers, as well as the challenges related to these formulations.

## 1. Introduction

Natural gums derived from plants and bacteria reveal numerous interesting properties potentially useful in various medical and pharmaceutical areas, including tissue engineering and drug delivery. In all biomedical applications, biocompatibility, biodegradability and low toxicity are crucial to developing novel materials useful as drug carriers or scaffolds for implanting cells in regenerative medicine. Most polysaccharides of natural origin are known as non-toxic and highly biocompatible materials with the ability to form porous hydrogels, being an excellent medium for cell growth and differentiation, as well as the incorporation of active ingredients. On the other hand, they are usually considered as cost-effective materials, as they occur abundantly in plants and are relatively easy to obtain. Polysaccharides are also very versatile materials and they can be easily modified, either by chemical modification or by physical addition of some other material. In this way, novel materials with interesting characteristics can be obtained. Among the most commonly applied procedures is crosslinking, leading to physical or chemical gels with altered properties compared to the original compound [1]. Crosslinking may be performed for a single compound and also for mixtures of different chemical entities. In such cases, the properties of the product are intermediate between the properties of the original compounds. Natural gums can also be combined with a wide range of natural and synthetic polymers belonging to other chemical groups which may also be a useful tool to design the materials with some specific characteristics [2]. An appropriate chemical and/or physical modification may lead to so called ‘smart materials’ which have the ability to change some of their properties as a response to an external stimuli, including temperature, pH or ionic strength change. These materials are particularly interesting from a pharmaceutical point of view, as they can be utilized as drug carriers releasing active ingredients upon contact with physiological conditions [3]. However, despite the numerous advantages displayed by natural gums, there are also some important drawbacks and challenges related to their application. For example, as natural products, gums can be synthesized by various organisms and the exact chemical structure, as well as molecular mass and physicochemical properties may differ depending on the origin of the compound which can potentially be a cause of repeatability issues. On the other hand, gums obtained from natural sources, such as plant seeds or exudates, usually contain some other compounds including proteins and minerals. The content of these minor components can vary depending on the source or manufacturing process parameters, which can also affect the properties of the product. It must be emphasized that the purity of both active ingredients and excipients is crucial for the quality of pharmaceutical dosage forms and the presence of potential impurities or difficulties encountered in the purification process can limit the usefulness of polysaccharide gums obtained from natural sources in large-scale industrial drug manufacturing. Finally, susceptibility to microbial contamination is mentioned among the drawbacks of polysaccharide gums [4].

Natural gum-based hydrogels can be useful as macroscopic networks and also as various particulate forms, including micro- and nanoparticles (micro- and nanogels, respectively). In pharmaceutical technology, these particles can be applied both in hydrated and dried form. The latter is usually administered orally as hydrophilic matrices, swelling upon contact with physiological fluids excreted in the gastrointestinal tract (GIT) and transforming into their original hydrogel state. Depending on the exact matrix composition and properties, the drug may be released at different parts of the GIT. It is important to note that most of the natural gums utilized in pharmaceutical technology, such as guar or xanthan, are not digested by the enzymes in stomach and duodenum, but they are substrates to glycosidase enzymes produced by the colon microbiome. The matrices prepared with the use of the polymers displaying the mentioned properties can pass the upper parts of the GIT without physical and chemical decomposition and release the drug in the colon, upon contact with bacterial enzymes. This important property made the natural polysaccharides an object of extensive investigations aiming at the development of novel dosage forms for targeted drug delivery to colon [5,6]. As unmodified gums usually reveal unfavorable mechanical properties and may intensively, swell which may lead to physical matrix erosion in the upper parts of the GIT, usually various crosslinking procedures or combinations with water-insoluble matrix-forming agents must be employed [7]. In this way, drug release kinetics can also be modified in order to obtain the desired dosage form characteristics.

In this review, the most recent scientific investigations regarding the application of natural gum-based micro- and nanoparticulate systems in drug delivery are presented. Considering the increased interest in the development of novel drug carriers with improved physicochemical characteristics, special attention has been paid to the chemical and physical modification of naturally occurring compounds. As the oral drug delivery route remains the most popular and convenient, this manuscript also covers dried hydrophilic matrices forming hydrogel particles upon contact with physiological fluids. The main objective of this article was to present the most recent research directions, focusing on natural polysaccharide gums and their derivatives applied as excipients in micro- and nanogel drug carriers. The literature studies summarized in this manuscript indicate that both natural gums and their derivatives are versatile compounds displaying tunable properties that can be tailored according to individual needs. On the other hand, gums are usually cost-effective materials with environmentally friendly features, which is a great advantage over synthetic polymers also frequently utilized in pharmaceutical technology.

## 2. Natural Gums

**Figure 1:**
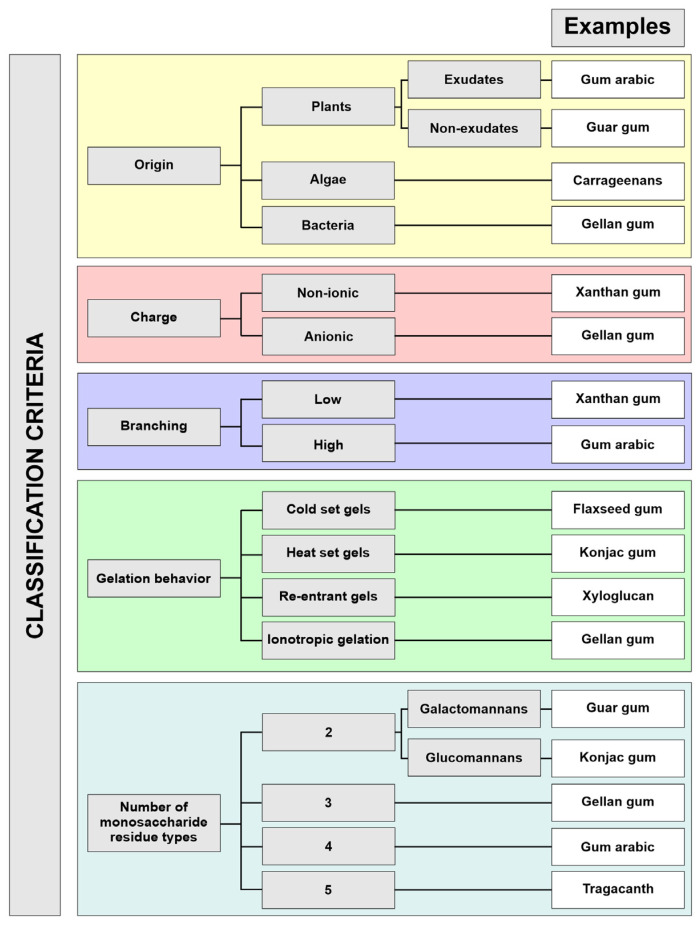
Natural gum classification according to different criteria [10,15].

The term ‘gum’ is used to describe natural polysaccharides displaying the ability to form viscous solutions or gels upon contact with water [8]. Depending on the origin, gums can be classified as microbial, seaweed- and plant-derived compounds. Gums obtained from plants can be further divided into exudate compounds, which are produced as a result of a mechanical injury or bacterial infection in a process called gummosis, and non-exudate compounds, which can be extracted from seeds or other plant tissues. Taking into consideration the chemical composition of gums in general, it is obvious that this class of compounds is wide and structurally diverse. In general terms, they can be considered as heteroglycans with different composition schemes, ranging from two monosaccharide unit types, such as in galactomannans, to more complex systems, such as gum ghatti or tragacanth, which are penta-heteroglycans [9,10]. It is noteworthy that the ratio of particular components, as well as the degree of backbone branching, can be important for the physicochemical characteristics, including the swelling ability of the gum [11]. Moreover, the particular compounds may have non-ionic properties, such as xanthan and guar gums, or carry a negative charge in the molecule, like gellan and karaya gums. The structural differences may also be related to different gelation behaviors, e.g., gellan gum reveals the ability to interact with divalent and trivalent cations, known as ionotropic gelation [12], xanthan gum gel formation ability is not temperature dependent [13] and konjac gum requires the presence of alkaline environment and heating [14]. The classification of naturally occurring gums according to different criteria is presented in Figure 1.

Natural gums have been used as excipients in pharmaceutical formulations for centuries. The most important example of natural polysaccharides employed as an excipient in pharmaceutical formulation is gum arabic, known for over 5000 years [16]. Taking potential pharmaceutical applications into consideration, the most important physicochemical property of natural gums is their ability to absorb water and swell. Because of this specific feature, they have been popularly used as binding and disintegrating agents in oral solid dosage forms. In liquids and semisolids, they are frequently used as thickeners and emulsifiers, as their ability to increase the viscosity of the product may be helpful to prevent sedimentation in suspensions or physical destabilization in emulsions. In solid dosage forms, they have been successfully used as excipients prolonging the drug release from tablets and other solid dosage forms, as they form gels decreasing the drug diffusion rate. Gums can also be applied to extend the drug release in other dosage forms, as it was shown for the marketed product Timoptic-XE^®^ (Merck; Rahway, NJ, USA), which was proposed as an alternative to a conventional low-viscosity formulation administered twice a day. Gellan gum applied as an excipient in the innovative product interacted with cations in tear liquid and formed a gel upon administration. In this way, the release of the active ingredient was extended, which allowed for the reduction in dosing frequency and also reduced the systemic exposure to the drug that, in this case, is supposed to act only locally [17]. Moreover, natural gums can play an important role in mucoadhesive formulations, enabling the formation of binding forces between the drug carrier and mucous membranes. This approach allows for extending the formulation residence time at the administration site and therefore improves the bioavailability of the active ingredient. Mucoadhesive formulations are usually administered to oral [18], vaginal [19] and nasal [20,21] mucosa but there are also examples of particulate formulations [22] or tablets [23] administered orally and adhering to gastric mucosa. However, in the case of formulations aiming at the mucoadhesive effect, usually chemically modified gums with thiol groups introduced to the structure are obtained and investigated [24,25,26,27]. Thiol groups can interact with cysteine occurring in mucin, which results in covalent bond formation and strong attachment to the surface of mucous membrane. In the case of non-modified polysaccharides, only relatively weak hydrogen bonds can be formed, which is also related to weaker bioadhesive properties.

Polysaccharide-based particulate systems are widely investigated as drug carriers in numerous areas of pharmaceutical and biomedical research. Micro- and nanoparticles obtained with the use of natural gums and their derivatives are employed for different purposes, regarding the diameter ranges. Nanoparticles are most commonly investigated as innovative drug carriers in injectable dosage forms, as nano-sized particles can be safely transported by the bloodstream without the risk of clogging the capillary vessels. The most extensively investigated areas are related to different anticancer approaches, including also targeted drug delivery. The modification of the particle surface may contribute to the selective uptake of nanoparticles by some specific cells, i.e., cancer cells. Polysaccharides can also be used as a component combined with some other material—for example, with gold nanoparticles [28] or mesoporous silica [29]—to modify the properties of the final product and adjust them to the individual needs. Moreover, nanoparticle-based systems can also be applied using a theranostic approach, allowing for both imaging and drug delivery. For this purpose, nanoparticles must be designed in a specific way, with both imaging and active pharmaceutical ingredients incorporated in the structure either covalently or physically [30]. It is also noteworthy that due to their neutral character, polysaccharide biopolymers are also excellent media for genes, nucleic acids and peptide delivery in the form of nanoparticles [31]. Finally, natural gums can be employed to obtain nanoparticles revealing specific surface-active properties allowing for selective adsorption at the water–oil interface in emulsion systems. In this way, solid nanoparticles can be used instead of classical surfactants and the systems stabilized in this way are known as Pickering emulsions. Pickering stabilizers must be insoluble in both phases and display similar wettability in polar and non-polar environment. Polysaccharides can be easily modified or combined with other materials in order to obtain the properties necessary to adsorb selectively in the interfacial area of the emulsion system. Pickering emulsions have gained a lot of attention recently, as they have good stability, can incorporate relatively high amount of the internal phase and are also free of the safety concerns usually raised whenever conventional surfactants are applied [32].

Scientific investigations involving gum-based particles in drug delivery focus on different areas and the obtained systems can be designed differently and administered via different routes. Microparticles are less frequently analyzed as potential injectable systems but are rather considered for oral drug delivery. Depending on the exact matrix composition, gums are usually applied in order to extend the drug release process or to move the drug release to the distal part of the gastrointestinal tract, which can be useful in the treatment of colorectal cancer or ulcerative colitis, as well as in chemopreventive approaches. It is noteworthy that such microparticles are usually used in a dried form, which swells and forms microgels upon contact with gastrointestinal fluids. Natural gums are particularly interesting in terms of oral drug delivery, as they reveal susceptibility to the enzymes produced by the microbiome in colon. Therefore, they are good candidates as matrix-forming agents in colon targeting. Polysaccharide-based particulate systems are also taken into consideration as potential protective agents allowing for the delivery of active ingredients sensitive to gastrointestinal enzymes or acidic environment of the stomach, such as insulin [33]. Another research direction focusing on gum-based microparticles is related to inhalable systems with potential application as an alternative therapy in tuberculosis. In such cases, the microparticles are also applied in a dry form and the particle size is crucial to reach the pulmonary area and exert the desired effect [34]. Nanoparticles can be taken into consideration as drug potential drug delivery systems administered via different routes. As it was already mentioned, most of the natural gums discussed here are non-toxic and biocompatible, which means they can be utilized as injectable dosage forms. Nanoparticles can be functionalized in many different ways allowing for targeting specific cells by receptor-mediated endocytosis [35]. It is noteworthy that some polysaccharide gums, such as xanthan gum, can be naturally recognized by mannose receptor which may lead to the intensified cellular uptake [36]. Mannosylation is among the strategies employed in nanocarrier surface modification in targeted therapies [37].

## 3. Micro- and Nanoparticle Fabrication Methods

### 3.1. Gelation Mechanisms

**Figure 2:**
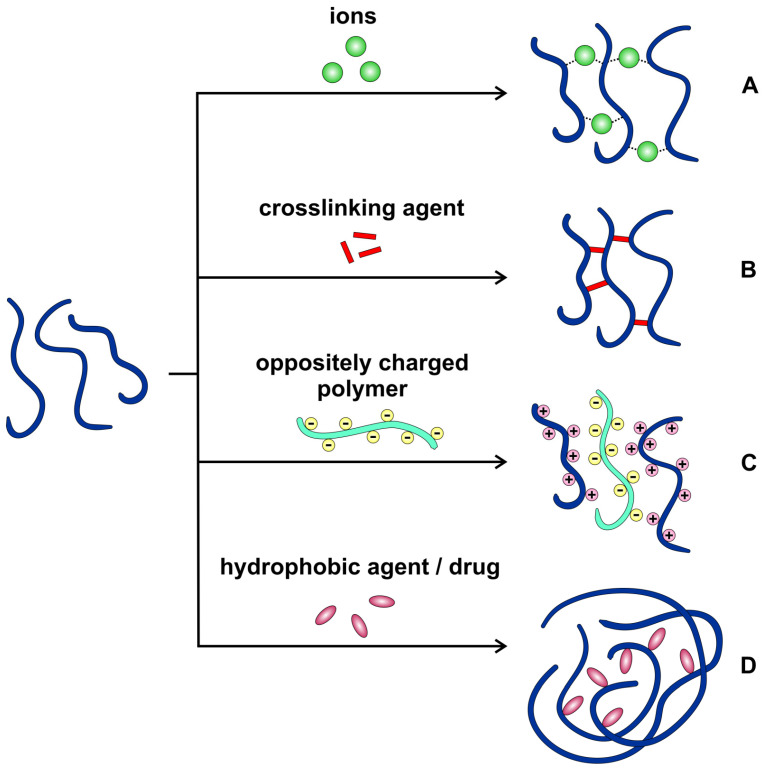
Mechanisms of gum-based micro- and nanoparticle formation: (**A**) ionotropic gelation, (**B**) covalent crosslinking, (**C**) polyelectrolyte complexation, and (**D**) drug or hydrophobic agent/polymer conjugation with self-assembly [31,38,39].

In order to form micro- or nanoparticles with the use of the described biopolymers, different manufacturing strategies are employed, depending on the chemical structure and physicochemical properties of the particular gum. The basic mechanisms underlying the particle formation, with the most important examples of natural gums are presented in Figure 2. It must also be emphasized that the preparation method may affect the properties of the final product, which is of crucial importance whenever the drug release kinetics is taken into consideration.

#### 3.1.1. Ionic Crosslinking

**Figure 3:**
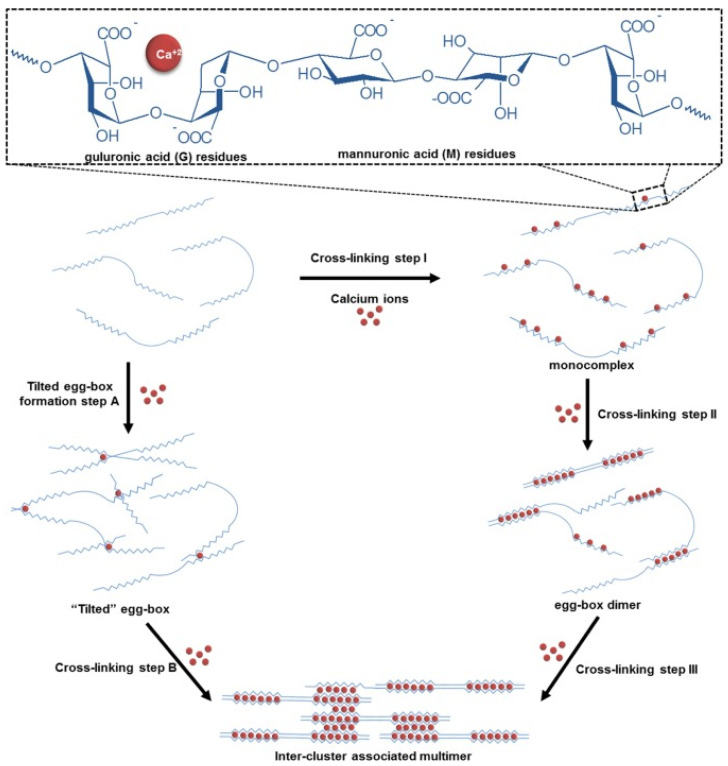
Schematic diagram showing the steps involved in calcium ion-induced crosslinking of the alginate (right, steps I–III) as suggested by Fang et al. [40] and (left, steps A and B) Borgogna et al. [41]. Reproduced from [45] with permission.

The ionic crosslinking process is typical for some gums that can interact with di- or trivalent cations, such as gellan gum or alginate. The cations interact with negatively charged parts of polysaccharide, forming bridges and thus initiating the formation of a gel structure. The active ingredient is entrapped between the crosslinked polysaccharide chains. It should be noted that the process is reversible and the applied crosslinking agents are usually considered as mild, which makes the obtained products fully biocompatible. In the case of alginate, the sodium ions are usually replaced with calcium cations and the gel formation process consists of a few steps. In the initial stage, the monocomplexes are formed; in the further steps, they transform into dimers and multimers, which is commonly known as the egg-box model [40]. According to another theory [41], the crosslinked polymer chains forming the dimer are not aligned in a parallel way but are approximately perpendicular to each other. In this way, a tilted egg-box structure is formed. The scheme depicting the formation of the mentioned structures is presented in Figure 3. Similar properties are widely described for gellan gum [42,43,44]; however, the whole process is more complex, as it is also temperature dependent. The polymer dissolves in water and forms random coils at temperatures exceeding 40 °C. Upon cooling, random coils transform into helices, which further aggregate into a reversible gel structure. The addition of cations induces the formation of ionic bridges between the helices and the aggregates formed in this way are stronger and do not undergo the reverse reaction, as it is seen for the ones obtained as the result of cooling alone [43]. Moreover, gellan gum occurs in low-acyl and high-acyl forms, which also affects the gelation process [12].

#### 3.1.2. Covalent Crosslinking

In covalent crosslinking, strong bonds bridging the adjacent polymer chains are introduced. The process is irreversible and the obtained junctions are generally insensitive to pH changes [38]. The applied crosslinking agent must contain at least two functional groups which can interact with hydroxyl groups in gum molecules. Among the most commonly utilized compounds, glutaraldehyde and sodium trimetaphosphate are mentioned [39]. It is important to note that dialdehydes are considered as relatively toxic, which limits their applicability in pharmaceutical technology. Alternatively, other less harmful crosslinking agents have been proposed, including genipin [46] or citric acid [47].

#### 3.1.3. Polyelectrolyte Complexation

**Figure 4:**
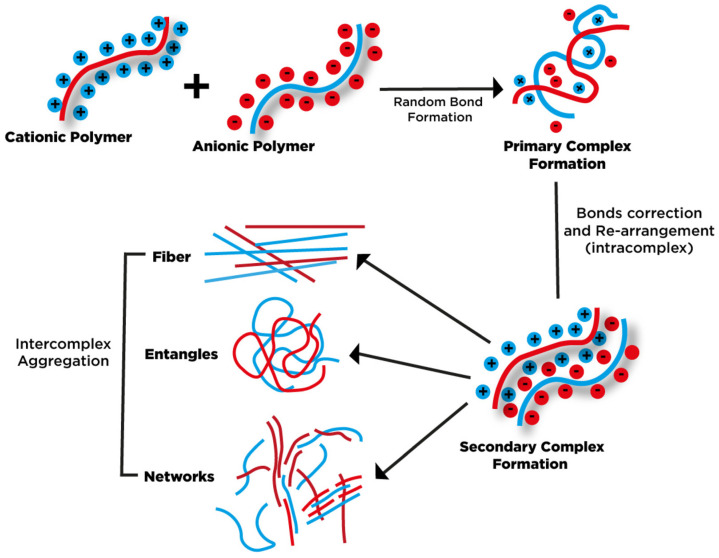
Illustration of the formation of polyelectrolyte complex. Reproduced from [48].

In this method, sometimes also described as complex coacervation, two oppositely charged polymers are used. As the negatively charged residues in one polymer interact with positively charged ones in the other polymer, the gelation process occurs as a result of complex formation. The properties of the product depend strongly on the ratio of cationic and anionic groups; and in the case of non-stoichiometric systems, the product is water soluble. Other important factors affecting the characteristics of the resulting product are pH and the mixing sequence of the particular components [48,49]. The scheme depicting the formation of polyelectrolyte complex is presented in Figure 4. As can be concluded from the available literature studies, natural gums are most commonly coupled with chitosan as a polycationic compound [50,51,52,53]. The process may be employed for the production of both solid microspheres and also for microcapsules with liquid core, when the complex is deposited on the surface of oil droplets and forms microcapsule shell [54,55,56].

#### 3.1.4. Self-Assembly

Self-assembling is observed in the case of modified gums with hydrophobic moieties introduced to the structure. In a polar environment, the amphiphilic molecule adopts spatial arrangement enabling hiding the hydrophobic moieties inside the particle, while the hydrophilic domains remain at the surface, serving as an interface between the hydrophobic core and the polar solvent. The process is spontaneous due to the decrease in the interfacial energy [39]. As the inner area of the particle is hydrophobic, the obtained systems may be useful in the delivery of active ingredients revealing low polarity [57,58,59]. In hydrophobic modifications, cholesterol [60], fatty acids [61] and other compounds [57,59,62] can be used. Parameters such as the particle diameter, the zeta potential and the loading efficiency can be modulated by adjusting the polymer chain length and the size of the hydrophobic residues [39].

#### 3.1.5. Polysaccharide/Drug Conjugation

In this approach, instead of a pharmacologically neutral moiety, drug molecules are covalently attached to the polysaccharide backbone. It is important to notice that in order to obtain a pharmacological effect, the bonds connecting the active ingredient and the carrier gum must be hydrolyzed and the drug must be released in its original form. Therefore, a biodegradable linker can be applied to connect both components of the conjugate. The properties of the linker can be cleaved in some specific conditions, which may be particularly useful in targeted cancer therapy [63]. It is also important to note that in the case of hydrophobic active ingredients attached to the hydrophilic gum backbone, the same spontaneous process of self-assembling takes place [64].

## 4. Gum-Based Micro- and Nanoparticles in Drug Delivery

### 4.1. Gum Arabic

Gum arabic (GA) is an amphiphilic polysaccharide [53,65] derived from dried sap of *Acacia* trees. Its molecule is highly branched and consists of D-galactose, D-glucuronic acid, L-rhamnose and L-arabinose. It possesses a lot of carboxyl groups that are mostly ionized at neutral pH, which is the reason why the molecule is extremely charged and has an expanded structure [65].

Gum arabic is soluble in cold water and the viscosity of gum solution is lower than other gums, only becoming viscous at high concentrations (30–50%). It is stable in acidic conditions and simultaneously its solution is slightly acidic (pH 4.5–5.0) with Newtonian flow [65]. When pH is above 2.2, GA is negatively charged due to the dissociation of carboxyl groups in its molecular chains [53]. It is a good stabilizer for oil in water emulsions, and displays good stability in a wide pH range, high ionic strength and high temperature stability [53]. Gum arabic possesses important functional features that make it very useful as an emulsifier for oils and flavors, texturizing agent, foam stabilizer, coating agent and encapsulating agent (especially for flavors) [65]. Studies have shown that gum arabic could form micro or nanocomplexes which may be useful as vehicles for carrying active ingredients in food and medical areas [53,66,67].

**Figure 5:**
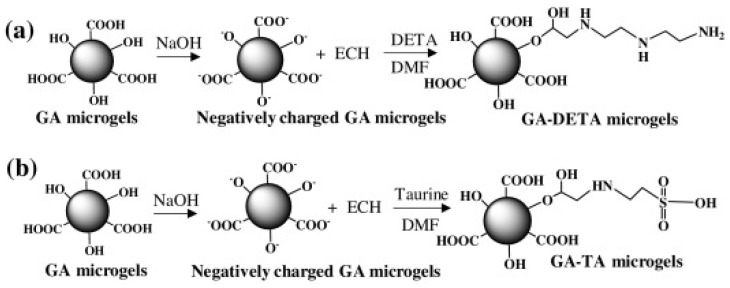
Schematic representation of the modification of GA microgels by (**a**) DETA and (**b**) TA. Reproduced from [66] with permission.

The properties of polysaccharides can be used for the preparation of hydrogels. Gum Arabic, because of its properties, can also work as a hydrogel former. An interesting investigation concerned with the use of gum Arabic was proposed by Farooq et al. [66], who evaluated microgels prepared with this polysaccharide. They used the reverse micellization method for the formulation of microgels with divinyl sulfone (DVS) applied as a crosslinker. In the study, a high yield was observed (78.5 ± 5.0%) and GA microgels prepared with this technique had superficial hydroxyl groups which gave a −27.31 ± 4.2 mV overall surface charge. The microgels were checked for degradation in different pH. As was demonstrated, at pH = 1, 22.8 ± 3.5% of microgels degraded hydrolytically during 20 days, whereas degradation was not observed at pH 7.4 and pH 9 at 37 °C. Further investigation was concerned with the influence of the modification of the structure on the zeta potential. Researchers used diethylenetriamine (DETA) and taurine (TA) as chemical modifying agents (Figure 5), GA microgels were chemically modified as GA-DETA and GA-TA, and the zeta potential values of 5.2 ± 4.1 and −24.8 ± 1.3 mV were measured, respectively, in comparison to −27.3 ± 4.2 mV for GA. Moreover, the blood compatibility of modified and non-modified microgels was tested via in vitro protein adsorption, the % hemolysis ratio, and the blood clotting index. Experiments showed that all microgels were hemocompatible, with a % hemolysis ratio between 0.23 and 2.05, and the GA microgels were found to be highly compatible with a blood clotting index of 81 ± 40. Moreover, all three microgels had low apoptotic and necrotic indices up to 50 g/mL, suggesting great potential in biomedical applications. Furthermore, the protonated DETA-modified GA showed a pronounced antimicrobial effect against *E. coli* and *S. aureus.* All received results indicated that microgels prepared with gum arabic and chemically modified microgels could be used as biodevices for potential biological applications with their tunable chemical and physical properties [66].

In their investigation, Zhou et al. [67] used gum arabic and also other polysaccharides such as carboxymethyl cellulose (CMC), alginate, pectin and carrageenan as the components for the preparation of egg yolk proteins/polysaccharide nanogels. They characterized the prepared particles for physical-chemical properties and also checked stabilities under simulated gastrointestinal conditions (SGI). In the investigation curcumin was used as the model lipophilic compound. The examination was performed for four curcumin/protein mass ratios, i.e., 0.5%, 1%, 1.5% and 2.5%. The evaluation was based on the encapsulation efficiency and loading capacity, also molecular interactions were described. Finally, the release of curcumin from protein-based nanogels was investigated by a dialysis method. Nanogels were spray-dried to obtain ultra-fine nanogel powder. The morphology and homogeneity of dry powders were studied by scanning electron microscopy (SEM) and the effects of used polysaccharides on these properties were described. The investigation showed that the polysaccharides were all able to form complexes with egg yolk lipoproteins. The obtained nanogels had a uniform particle size below 85 nm and displayed zeta potential >30 mV. Evaluated nanogels prepared with all polysaccharides received similar encapsulation efficiency and loading capacity [67]. The release study showed that dissolution in the gastric phase of free curcumin reached 76% during the 120 min. It was much more rapid compared to the release from nanogels. The fast diffusion of free curcumin was continued after transfer to simulated intestinal fluid and was complete in 210 min. All investigated nanogels showed a sustained-release profile of curcumin both in gastric and intestinal pH. Complexation with pectin and gum arabic gave moderate and similar prolonged release of curcumin in acidic condition, with approximately 55% of curcumin released. During incubation at pH 7.5, the nanogel with pectin showed a slower release than the nanogel with gum arabic. Among all three types of investigated nanogels, complex with CMC demonstrated the slowest kinetic release and the smallest accumulative release in both used conditions (gastric and intestinal).

Another interesting study on the use of gum arabic and curcumin was proposed by Han et al. [53]. The authors studied the encapsulation of curcumin in Pickering emulsion and applied nanoparticles containing a complex of chitosan (CS) and gum arabic (CS/GA) as an emulsion stabilizer. The emulsifying properties of CS/GA nanoparticles were evaluated and the stability and curcumin release performance of CS/GA nanoparticle-stabilized Pickering emulsion was studied. The first step was the preparation of nanoparticles with the ratio CS:GA 1:5 with a dripping method and the precipitate was freeze dried. The next step was preparation of the CS/GA nanoparticle-stabilized Pickering emulsions in which the water–oil ratio was fixed at 0.5. The dispersions were controlled in different nanoparticle concentrations (0.15%, 0.30%, 0.45%, 0.60% and 0.75% w/v). Additionally, the effects of pH (3.0, 4.0, 5.0, 6.0, and 7.0), ionic strength (0, 100, 200, 300, and 400 mM) and temperature (−4, 25, 37, and 60 °C) on the stability of the emulsions were investigated. Experiments confirmed that chitosan with a positive charge can interact with gum arabic with a negative charge to form polyelectrolyte complexes and CS/GA composite nanoparticles were successfully prepared. Additionally, interactions between CS and GA were confirmed by FTIR and XRD examinations. With the fluorescence microscopy researchers observed that nanoparticles were aggregated at the water–oil interface to stabilize prepared Pickering emulsion. Additionally, the stability of emulsion significantly increased with the nanoparticle concentration. Studies showed that CS/GA nanoparticles stabilized Pickering emulsion to temperature variation but it was sensitive to pH and ionic strength. Additionally, curcumin encapsulated in the CS/GA nanoparticle-stabilized Pickering emulsion showed a low degradation rate during storage. Furthermore, the obtained system displayed a sustained release of curcumin. These results confirmed that the CS/GA nanoparticle-stabilized Pickering emulsion could be used as an effective delivery system for bioactive substances.

Gum arabic, after structural modification, could also be used as the crosslinking agent in the preparation of nanogels. Such a possibility was proposed by Sarika and James [68], who prepared gelatin–gum arabic aldehyde (Gel–GAA) nanogels by an inverse mini-emulsion technique. The formulation of nanogel was based on the preparation of two separate emulsions, where one contained gum arabic aldehyde nanoparticles, while the other contained nanoparticles of gelatin. Prepared emulsions were mixed together and sonicated to obtain a Gel–GAA nanogel. The received particles were then dried under reduced pressure to obtain the nanogel powder. The analysis of the morphology of the particles in emulsion, as well as after redispersion, confirmed the nanometric size and the spherical morphology of the particles. In vitro cytotoxicity analysis proved the non-toxic nature of the nanogels and also the hemocompatibility analysis indicated that the nanoparticles could be useful in drug delivery.

### 4.2. Guar Gum

**Figure 6:**
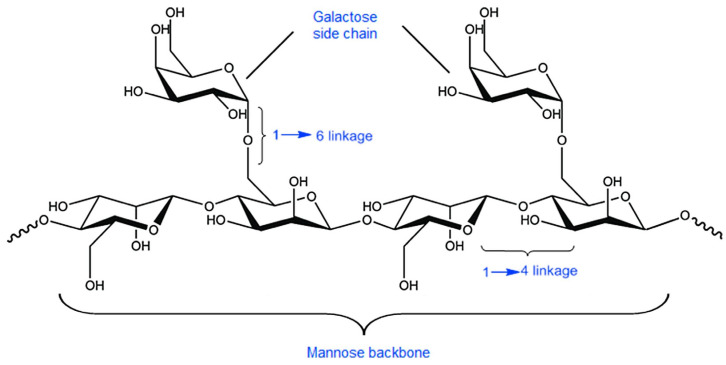
Typical structure of guar gum. Reproduced from [70] with permission.

Guar gum is obtained from the seeds of *Cyamopsis tetragonolobus*, which belongs to the *Leguminosae* family. The main component extracted from the plant material is non-ionic galactomannan with the main backbone composed of β-d-mannopyranosyl moieties connected by 1,4-glycosidic bonds. The side chains are α-d-galactopyranosyl units attached to the backbone with 1,6-glycosidic bonds (Figure 6). It is widely applied as a thickener and stabilizer in a few industrial areas, including paper, food, explosives and textiles manufacturing [69]. Considering the potential pharmaceutical utility of the compound, its most important physicochemical features should be mentioned. Guar gum is strongly hydrophilic and forms shear-thinning systems upon hydration without heating [70]. Because of the non-ionic properties of the polysaccharide, the obtained dispersions are stable in a wide pH range [71]. Moreover, the compound is a substrate for bacterial enzymes in the colon. Therefore, guar gum is extensively investigated as a binding and disintegrating agent in solid dosage forms, as well as a matrix-forming agent in sustained-release products and a carrier in colon-targeted drug delivery systems [72,73,74,75]. Guar gum also displays the ability to interact with mucin, which makes it useful in the development of mucoadhesive formulations [76]. The native form of guar can be easily modified to alter its properties by esterification [77], sulfation [78] and etherification [79] of hydroxyl groups. Moreover, the compound can be crosslinked [80] or grafted with another polymer [81].

Considering microparticulate guar gum-based systems, the most extensively studied area is related to the development of novel drug delivery systems designed to deliver the active ingredient to the colon. Sharma et al. [82] prepared microparticles with embelin, plant-derived benzoquinone derivative displaying anti-inflammatory, antioxidant and wound healing properties as an alternative therapeutic approach in ulcerative colitis. The microparticulate system was prepared with the use of water-in-oil emulsion with gum dispersed in an internal phase. The water and oil phases also contained appropriate surfactants (Tween^®^ 80 and Span^®^ 80, respectively; Loba Chemie, Mumbai, India). In order to obtain microgel particles, the polymer was crosslinked with glutaraldehyde. The product was dried and tested for physicochemical properties, including particle size, drug entrapment efficiency and storage stability, as well as for in vitro drug release and biopharmaceutical properties, which was performed with the use of Wistar rat models. It was found that the entrapment efficiency was affected by the concentration of the surfactant in the oil phase, as it increased miscibility of both phases and solubility of the drug in oil. In an in vitro study, it was demonstrated that only a small amount of the incorporated drug was released in the acidic medium simulating gastric fluid, which is promising in terms of moving the activity of the product to the distal parts of the gastrointestinal tract. In vivo studies revealed that pretreatment with the investigated drug prevented the development of colitis in experimental animal models. Moreover, the analyzed dosage form showed similar results as mesalamine, a standard therapeutic agent applied in colitis ulcerosa. It was also found that embelin entrapment in microparticles improved its efficacy when compared to free drug administered orally.

**Figure 7:**
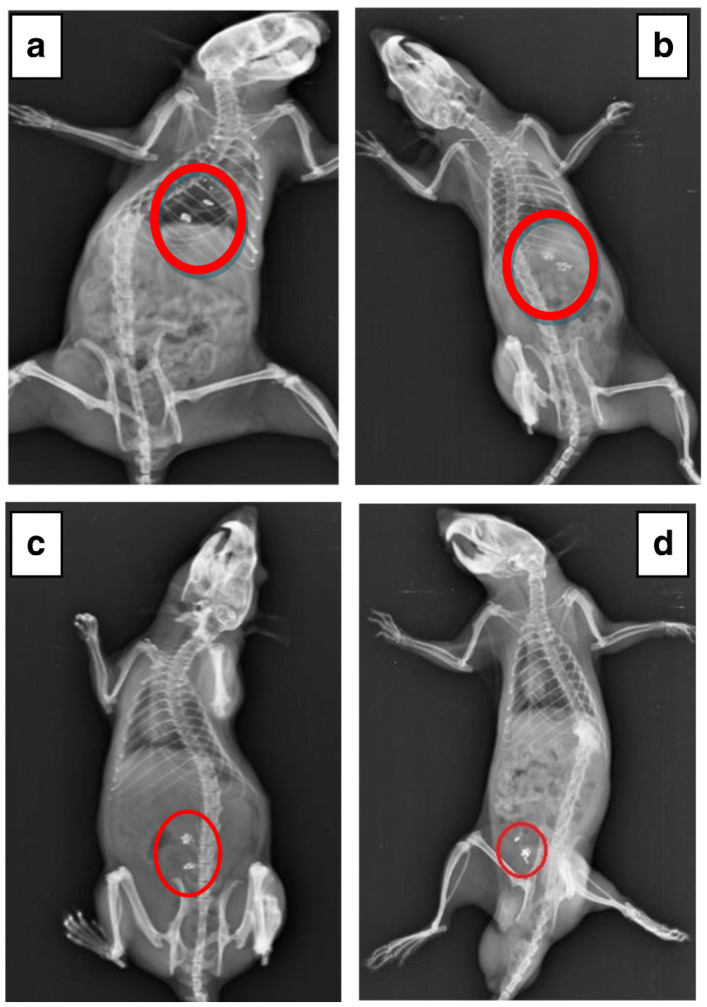
X-ray images of rats treated with barium sulfate-loaded microparticles obtained after (**a**) 2, (**b**) 4, (**c**) 6, and (**d**) 6 h. The red circles indicate the presence of BaSO_4_-loaded microparticles. Reproduced from [75] with permission.

A similar preparative approach was applied by Kang et al. [75] to form a microparticulate system with doxorubicin and metformin hydrochloride for potential application in colorectal cancer. The obtained product was also tested for physicochemical properties, in vitro drug release and in vivo efficacy in an animal model. The obtained particles were uniform, displayed a diameter of approximately 15 μm and showed only slight evidence of a physical interaction between guar gum and metformin hydrochloride, as demonstrated in X-ray powder diffraction (XRPD) and differential scanning calorimetry (DSC) experiments. The drug release study performed in three different media, i.e., simulated gastric, intestinal and colonic fluids, revealed that the majority of the drug was released in conditions similar to the physiological environment of the colon. Moreover, the third stage of the drug release experiment was performed with or without the addition of rat cecal contents and the comparative analysis indicates that the drug release rates were significantly higher in the presence of colonic enzymes which could be related to the alteration in guar gum matrix swelling and erosion. The cytotoxicity studies performed with the use of human epithelial colorectal adenocarcinoma cells revealed that combined doxorubicin and metformin microparticle-based treatment was more effective than doxorubicin solution but less effective than combined doxorubicin and metformin solution. It was also shown that the analyzed formulation performed better in terms of tumor regression in vivo compared to doxorubicin solution and combined doxorubicin and metformin solution. It is also noteworthy that the X-ray images obtained for the animals treated with barium sulfate-loaded microparticle analog of the analyzed formulation confirmed its ability to reach the colon without visible signs of physical decomposition in the upper parts of the gastrointestinal tract (Figure 7).

Another approach was presented by Hu et al. [83], who prepared heterogeneous microgels with a core composed of polyvinyl alcohol (PVA) and guar gum and a shell containing alginate and chitosan bound to each other with electrostatic interaction. The described microgels were loaded with curcumin for potential application in ulcerative colitis. In the first step, water-in-oil emulsion with guar gum, PVA and calcium chloride dispersed in an internal phase was prepared. Next, the emulsion was mixed with an alginate solution, which interacted with CaCl_2_ in the emulsion droplets, forming a shell. The obtained beads were subsequently placed in chitosan solution to form another layer surrounding microgels. In the last step, the product was frozen and thawed for a few cycles and the obtained particles were dried. The aim of the applied procedure was to effectively incorporate curcumin, which is commonly known for its extremely low solubility in water. Moreover, the formulation was designed to avoid drug release in the upper parts of the gastrointestinal tract and to deliver it to the colon. The obtained product was tested for swelling behavior at three different pH values corresponding to the conditions in different parts of the gastrointestinal tract, i.e., stomach, intestines and colon. It was shown that in the simulated gastric fluid, the swelling rate was very low and increased significantly in simulated gastric fluid, after the outer chitosan layer was removed in acidic conditions and alginate was ionized. Moreover, it was found that the drug release was pH dependent as well. In simulated gastric fluid, the drug release was slow and the rate increased significantly both in simulated small intestine and colon fluids. Finally, pharmacokinetic parameters were evaluated based on the experiments performed with the mice model. The investigated formulation was compared to plain curcumin solution and it was shown that plasma concentration, as well as the concentrations in gastric, intestinal and colon tissues, were more stable in the case of microgels. The obtained results indicated that the analyzed system was suitable for sustained drug release products. It was also found that the symptoms of inflammation, including biochemical markers and histopathological alterations, were reduced in the case of animals treated with microgels. Interestingly, the formulation revealed also the ability to accumulate in colon tissue for a prolonged time, which was demonstrated based on fluorescence imaging experiments.

As was already mentioned, the properties of native gums can be altered either by chemical modifications or by blending with other polymers. Eswaramma et al. [84] described microgels prepared with the use of chitosan and guar gum with attached poly((2-dimethylamino)ethylmethacrylate) (PDMAEMA) side chains. The particles were loaded with 5-fluorouracil, an anticancer agent. PDMAEMA and its derivatives are known for pH responsiveness; therefore, in the presented study, it was applied to modify the characteristics of guar gum and chitosan. The manufacturing method was based on water-in-oil emulsion containing polymers dispersed in an internal phase. As a crosslinking agent, glutaraldehyde was used. It was demonstrated that the obtained product revealed higher swelling ability. This parameter was also higher in the case of lower crosslinking density. The obtained microgels revealed interesting pH-sensitive properties, as they had the tendency to swell more intensively at acidic pH values. It was concluded that this behavior resulted from the addition of cationic polymers, as guar gum alone did not reveal such features. The drug release test performed at pH = 1.2 and 7.4, corresponding to gastric and intestinal environments, respectively, showed that the drug release rate is correlated with swelling ability. The microgels containing grafted polymer and chitosan released the drug faster at pH = 1.2. The drug release rate was also generally higher in the case for complex formulation than for guar gum-based microparticles.

Another combined microparticulate formulation was described by Phadke et al. [85]. In the study, a carboxymethyl guar gum (CMGG) and gelatin blend was used to encapsulate theophylline, a phosphodiesterase inhibitor applied in the therapy of asthma. The particles were formed with the use of a water-in-oil emulsion system and the polymer was crosslinked with glutaraldehyde. Similarly to the previously mentioned studies, the particles were isolated and dried. The encapsulation efficiency tests revealed that the parameter decreased with the increase in CMGG content, as the polymer network became loose and the drug particles could migrate outside. The DSC experiments suggested that the active ingredient could be dispersed in the polymer matrix in an amorphous form, as no endothermic peak corresponding to theophylline melting was observed in the plot. This conclusion was also supported by the results of XRPD experiments. Swelling studies revealed that the rate of the process depended on the crosslinking density. The drug release study was performed at pH = 1.2 for the initial 2 h; and in the next stage, the dissolution medium was exchanged for pH = 7.4 buffer. The highest release rate was observed for the formulation with the highest amount of guar gum derivative which was most probably related to its high swelling ability. With the increase in crosslinking density, the drug release rate decreased. Analyzing the drug release profiles, it was concluded that the process followed the diffusion mechanism.

Apart from guar gum-based microgels, which are most commonly applied in a dried form and administered orally to restore their hydrogel form in situ, the polymer is also utilized to obtain various nanoparticulate forms. Dodi et al. [86] described carboxymethyl guar gum nanoparticles with Rhodamine B as a model compound. Carboxymethylation was used to improve the unfavorable properties of a native compound, including uncontrolled hydration and swelling rate and susceptibility to microbial growth. The particles were prepared with ionic crosslinking with sodium trimetaphosphate (STMP) as a crosslinking agent. The obtained particles were approximately spherical. The cytotoxicity study conducted with normal human dermal fibroblasts showed that the novel material was non-toxic at concentrations below 0.3 mg/mL. At higher concentrations, a gradual decrease in cell viability was observed. The presence of a model active ingredient affected the particle diameter, the zeta potential and the polydispersity index of the obtained particles. As expected, the diameter was higher in the case of the active-loaded samples. The zeta potential for all analyzed systems was negative; however, the absolute value recorded for the rhodamine-loaded system was lower when compared to placebo systems. The drug release study was performed in conditions simulating gastric and intestinal environments. It was found that only approximately 37% of the incorporated rhodamine was released in the acidic medium, while the release was almost complete at pH = 7.4. The obtained results indicated that the investigated system was pH-sensitive and could be potentially applied in modified release dosage forms.

Kaur et al. [87] described guar gum nanoparticles loaded with Ag85A antigen for potential oral immunization against tuberculosis. The aim of the study was to prevent the degradation of the antigen in an acidic environment of the stomach. It was also hypothesized that guar gum could be recognized by mannan receptors which would induce endocytosis and improve the cellular immune response. The system was obtained with nanoprecipitation method with ethanol as an antisolvent. In the last step, the formulation was spray dried. The obtained particles were spherical and displayed a diameter of approximately 900 and 1800 nm before and after drying, respectively. Only 12% of the antigen was released in an acidic medium in 4 h, while the release rate increased significantly in simulated intestinal fluid. The efficacy of the investigated system was also tested in vivo in mice. Histopathological studies and confocal microscopy imaging demonstrated that indeed the antigen incorporation in guar gum-based nanoparticles significantly increased its uptake from the intestines. It was also demonstrated that the animals administered with the nanoparticulate system presented a better immune response measured as IgG and IgA levels. The described results seem to be promising in terms of the development of novel oral alternatives to the most commonly applied injectable vaccines.

Liu et al. [88] obtained and characterized chemically modified guar gum with vitamin E derivative attached to the polymer backbone through a succinate moiety acting in this case as a linker. The modified polymer was utilized as a carrier for borneol, plant-derived bicyclic monoterpene displaying numerous activities beneficial in terms of the therapy of conditions localized in central nervous system. The compound has the ability to permeate blood–brain barrier; however, its applicability is limited because of poor solubility in water. The aim of the study was to design a dosage form that would efficiently deliver both active ingredients to brain cells, overcoming also the difficulties encountered in the case of poorly water-soluble actives. The chemical modification of the polymer resulted in self-assembled core-shell nanoparticles with the diameter of approximately 210 nm and spherical morphology. The obtained product revealed also good biocompatibility, as confirmed by in vitro cell toxicity studies and hemolysis assay. However, in the case of borneol-loaded particles, the results were slightly worse, which was most probably related to the properties of the active ingredient released during the study.

### 4.3. Alginate

Alginate is polysaccharide derived mostly from brown algae from *Phaeophyceae* class. The compound can also be synthesized by bacteria including *Azotobacter* and *Pseudomonas*, however, the bacterial products usually display high molecular mass, while in the case of plant-derived ones wide range of masses is observed. The term ‘alginate’ is commonly used in the studies regarding both alginic acid and its salts [89]. Alginates have a linear structure, with β-d-mannuronic (M) and α-L-guluronic (G) acid moieties occurring in the structure in different ratios and connected to the adjacent ones with 1,4-glycosidic bonds. The residues are organized in homopolymeric MM and GG blocks, composed of only one type of monosaccharide unit, and heteropolymeric blocks containing both [90] (Figure 3). Among the most important properties of alginate is its ability to interact with cations and form gels in a process commonly known as ionotropic gelation. It is noteworthy that only guluronate residues can participate in the formation of ionic bridges. Therefore, the length of GG blocks and the ratio between mannuronic and guluronic moieties are important in terms of physical properties of the polymer and its gelling ability. The polymers rich in G moieties produce gels revealing relatively high rigidity, while the ones with lower G content are softer and more elastic [91,92]. The exact composition of the compound and the properties of the obtained gels depend on the source of the compound [93]. The gelation process depends also on the applied cations and their binding to the polymer. In the case of divalent cations, the crosslinking strength is described in the following order: Mg^2+^ <<  Ca^2+ ^ <  Zn^2+ ^ <  Sr^2+ ^ <  Ba^2+^ < trivalent cations; however, in biomedical and pharmaceutical areas, usually calcium ions are applied for safety and economic reasons [90,94].

Alginate is widely applied as a food additive, pharmaceutical excipient and wound dressing material. Its most important advantages are excellent biocompatibility and biodegradability, as well as cost-effectiveness. In the research related to pharmaceutical technology and drug delivery, alginate can be used as a thickener, gelling, stabilizing agent [95,96], as well as tablet disintegrant [97]. As the polymer reveals the ability to interact with cations, it is an excellent material for obtaining various types of micro- and nanoparticles. Similarly to other natural gums, alginate is extensively investigated as a carrier for targeted drug delivery to distal parts of the gastrointestinal tract, which can be useful in innovative therapeutic approaches to the treatment of colorectal cancer [94], Crohn’s disease and ulcerative colitis. Ionotropic gelation does not require the application of any potentially toxic reagents and harsh reaction conditions associated with drug decomposition risk, which is the reason why this method is popularly applied to obtain various particulate systems, including polymer gels which are dried and applied as solid dosage forms. Obviously, the physical crosslinking agent should be also subjected to careful consideration. Ions such as Pb^2+^ which reveal good crosslinking properties are also toxic and cannot be applied in pharmaceutical formulations. Depending on the dosage form design and the individual needs, alginate can also be crosslinked covalently or both crosslinking methods can be combined in one formulation. The properties of native alginate can also be modified with the presence of another polymer.

Patole and Pandit [98] prepared mesalamine-loaded alginate-based microparticles, which were loaded to hydroxypropyl methylcellulose (HPMC) capsules coated with Eudragit^®^ FS-30D (Evonik Industries Ltd., Mumbai, India) to improve the resistance to acidic conditions of the stomach. The particles were prepared with the use of a water-in-oil emulsion crosslinking method and as a crosslinking agent CaCl_2_ isopropanol solution was used. In the drug release studies performed for dried beads alone in acidic conditions, burst release was observed, which is not favorable in the case of a formulation intended to exert therapeutic action in the colon. The observed effect could be related to the high porosity of the beads, the dissolution of the polymer in an acidic medium and an insufficient concentration of the polymer, which might lead to the increased contact of the incorporated drug with the dissolution medium. Therefore, the application of an acid-resistant capsule shell was necessary to deliver the drug to the colon. The drug release studies performed for capsules with the use of simulated intestinal fluid and simulated colon fluid with the addition of rat fecal content revealed a significant increase in the drug release rate in the latter case. The observed effect was related to bacterial degradation of alginate and confirms that drug incorporation into alginate matrix improves selectiveness towards the colon. In vivo studies performed for microspheres proved their efficacy in mice with induced ulcerative lesions.

**Figure 8:**
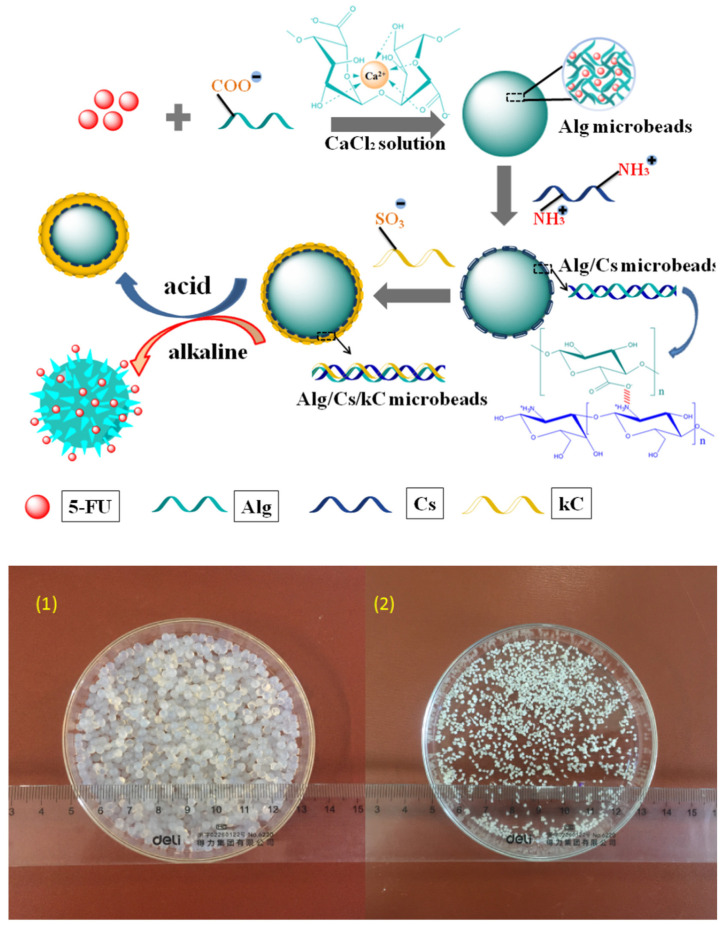
Scheme for the preparation of 5-fluorouracil-loaded alginate (Alg) microbeads with a chitosan (Cs) and carrageenan (kC) coating, and digital photographs of freshly prepared (**1**) wet and (**2**) dry microbeads. Reproduced from [99] with permission.

Sun et al. [99] presented alginate-based microparticles coated with chitosan and κ-carrageenan for potential colon-targeted release of 5-fluorouracil. The aim of applying additional coating layers was to obtain better control of the drug release along the gastrointestinal tract and to target its distal parts with better selectivity. The particle core was prepared with simple ionotropic gelation performed with calcium chloride. The coating layers were then applied sequentially by immersion in appropriate polymer solutions. As alginate is a negatively charged polymer, it forms electrostatic interactions with positively charged chitosan, which, in turn, can bind negatively charged carrageenan. The final product was separated and dried (Figure 8).

Three types of microbeads, i.e., uncoated, with one coating layer and dual-coated, were subjected to swelling studies performed in simulated gastric, intestinal and colon fluids. Interestingly, the highest swelling degree in an acidic medium was observed in chitosan-coated particles. The particles with dual coating swelled slower, while the lowest degree was recorded for plain alginate microbeads. The observed phenomenon was explained by the properties of chitosan, which becomes protonated in the presence of HCl. The process weakens the coating due to the repulsive interactions between protonated amine groups. On the other hand, the interaction between chitosan and alginate also becomes weaker. Because of the positive charge located on the surface of the particle, it becomes more hydrophilic and, therefore, more susceptible to water penetration. For all investigated systems, the swelling in intestinal and colon fluids was significantly faster. Plain alginate beads dissolved completely in an intestinal fluid, while both coated formulations behave similarly. The observed phenomenon is related to barrier function of chitosan layer which prevented physical decomposition of polymer matrix. The drug release was tested with the use of the same media as in swelling studies. It was found that all three formulations revealed the lowest drug release rate in acidic solution, which was related to both alginate and active ingredient pH-dependent solubility. The results observed in this analysis corresponded to those recorded in swelling tests; the highest drug release rate was obtained for chitosan-coated beads. In simulated intestinal and colon fluids, plain microbeads released most of the active ingredient within 4 h, while both coated formulations displayed lower release rates with the same trend as observed in swelling experiments. The described study shows that the properties of the matrix-forming agent can be easily and efficiently modified to obtain the pharmaceutical properties required in a particular medical condition.

**Figure 9:**
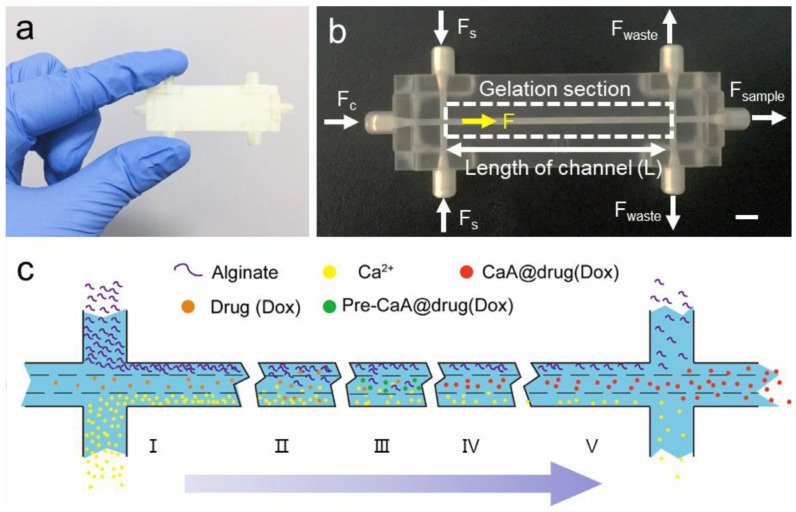
A 3D-printed concentration-controlled microfluidic chip for the synthesis of calcium alginate microgels and the diffusion mixing pattern. (**a**) Photograph of the 3D-printed concentration-controlled microfluidic chip. (**b**) Photograph of the 3D-printed concentration-controlled microfluidic chip of 30 mm. The F_c_ represents the middle stream flow rate. F_s_ represents the side stream flow rate. F represents the total flow rate in channels. F_waste_ the flow rate of the waste solution and F_sample_ is the flow rate of the sample. The scale bar is 3 mm. (**c**) Schematic representation of the diffusion mixing pattern where the large differential concentration drives the diffusion of alginate molecules and Ca^2+^ ions into the central stream, and later gradually generating microparticles when traveling along the microchannels. The formation of products is divided into the following steps: entrance (I), diffusion (II), mixing (III), gelation (IV), and collection (V). Reproduced from [100].

As was mentioned above, because of the simplicity, cost-effectiveness and non-toxicity of the applied reagents, ionotropic gelation is the most frequently utilized manufacturing technique in the case of alginate-based microgels. The most popular technique applied to obtain ion-crosslinked alginate is related either to instilling droplets of one solution into another or to preparing an emulsion. However, there are also some studies involving different techniques. For example, Cai et al. [100] prepared 3D-printed microfluidic chip to mix alginate and crosslinking solutions and in this way improve the control of the ionotropic gelation process (Figure 9). The chip was concentration-controlled and the particle size could be regulated with the length of the central channel in the chip, as well as a flow rate ratio. In the study, an efficient alternative method to obtain microgels loaded with doxorubicin was presented, with investigations regarding the impact of the chip construction features on the particles properties. In fact, the obtained placebo gel diameter was within a nanometer range (327–514 nm) while the drug-loaded particles were larger (388–1655 nm). The particles containing doxorubicin were also tested for the zeta potential, drug release and cytotoxicity towards epithelial cell lines applied in studies focusing on breast cancer. All performed studies indicate that the developed and described method can be useful to produce alginate particles with designed properties and to avoid some of the difficulties encountered with conventional ionotropic gelation methods. Microgel fabrication with microfluidic chips was also reported in other studies [100,101,102,103].

Volpatti et al. [104] prepared and described alginate microgels with incorporated insulin-loaded nanoparticles. Even though diabetes mellitus has been known for centuries and insulin-based treatment has been widely accepted and applied as a standard treatment, the control of glucose levels in diabetes-affected patients still remains a challenge. Currently, there are no glucose-actuated drug delivery systems in the global pharmaceutical market. The aim of the study was to obtain a system for self-regulating insulin delivery. Nanoparticles were composed of acetalated dextran, catalase, insulin and glucose oxidase. Their function was to respond to the changes in glucose concentration in the surrounding environment, while porous alginate microgels were used as an external protective matrix, also allowing for controlled release of the active ingredient. At high glucose levels, glucose oxidase transforms glucose into gluconic acid, which is associated with the decrease in pH in the microgel particle. This, in turn, leads to the hydrolysis of acetal bond in dextran derivative and in this way insulin is released to the surrounding media. The described microgels were obtained with ionotropic gelation performed with the use of electro-spray system. In order to check the effectiveness of the applied approach, the particles were loaded with FITC-insulin as a fluorescent probe and incubated in media with different concentrations of glucose. It was demonstrated that the amount of released active ingredient indeed depended on glucose level and the external alginate matrix protected insulin from degradation for over a week in vitro. The system was also tested in vivo with the use of mice model with good results. It was demonstrated that two 60 IU/kg doses of insulin incorporated in a novel formulation allowed for keeping the glucose levels in diabetic mice at normal levels for 22 days.

It is noteworthy that alginate microgels are frequently investigated as carriers for cell delivery in tissue engineering and regenerative medicine. However, these applications have been summarized and described elsewhere [105].

### 4.4. Gellan Gum

**Figure 10:**
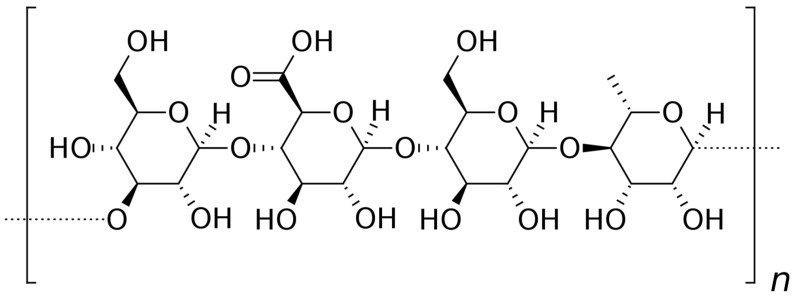
Structure of gellan gum.

Gellan gum was discovered in the lab of Kelco company, the division of Merck in 1978 [106]. It was originally isolated from the water plant of *Elodea* sp. That species turned out to be a non-pathogenic Gram-negative organism from the species of *Pseudomonas*. The currently applied name is *Sphingomonas elodea* [107]. The first applications of gellan were related to the replacement of agar in microbiological laboratories. Compared to the agar gel, gellan revealed beneficial features such as higher clarity, lower contamination level and lower concentrations required to keep the same mechanical properties. Currently, gellan is used as a food additive throughout the world as E418. Gellan gum is an anionic, water-soluble linear hetero-polysaccharide with high molecular weight of approximately 1.0 kg/mol. Its basic structural unit is a tetrasaccharide which consists of α-L-rhamnose, β-d-glucuronic acid and β-d-glucose, in the molar ratios of 1:2:1 [108]. In its native form, it has two acetyl groups, which can be removed by an alkaline hydrolysis resulting in obtaining its modified form—low-acyl gellan. The structure of gellan is presented in Figure 10.

Gellan gum is among the most promising exopolysaccharides and has lately gained much interest among researchers because of its ability to form a transparent solid gel in the presence of cations, which is called ionotropic gelation [109]. Among its most important properties, the lack of toxicity, biodegradability and biocompatibility should be mentioned. Furthermore, gellan gels are transparent, heat resistant, and pH dependent. Such properties make it a suitable candidate to use in the pharmaceutical and food industries as emulsifying, stabilizing, thickening and gelling agents and also in the preparation of controlled-released drug delivery systems [12]. Gellan can be used in semisolid as well as in solid formulations. Recently, it has also been tested for formulations of nano- and microgels for widespread applications. Gellan microgels obtained with crosslinked gellan for potential application in delivery systems were proposed by Vilela et al. [110]. Such formulation was produced by atomization followed by ionotropic gelation and coating with chitosan in order to improve its resistance to gastric juice. As a result, the authors obtained microparticles at a gellan concentration above 0.6% with an average size of 70–120 µm. The obtained formulation showed high stability in aqueous media. However, coating with chitosan enhanced the stability and prevented the disruption process. Furthermore, the performed digestion test showed the resistance of the formulation to simulated gastric conditions. Network disruption was, again, more intense for uncoated formulations. However, the tested formulation exhibited a high number of undamaged particles even after 2 h in simulated enteric juice. Hence, gellan-based microgels can be a suitable vehicle for delivery of bioactive compounds, and can be manufactured in mild conditions using a simple process and biocompatible ingredients. The combination of anti-inflammatory and anticancer agents seems to be promising in cancer therapy. Hence, D’Arrigo et al. [111] proposed a gellan gum-based nanogel containing prednisolone and paclitaxel. They obtained the formulation with prednisolone chemically bonded with a gellan, which resulted in a nanohydrogel formation. Paclitaxel, in turn, was physically entrapped in it as the hydrogel had a core-shell structure. A nanohydrogel system acted as a solubility enhancer of drugs, which resulted in improved drug performances, increasing the cytotoxic effect in vitro on several types of cancer cells. Mahajan and Patil [112] produced chitosan-gellan gum-based polyelectrolyte complex-based nanogels for curcumin delivery to cancer cells. Prepared formulations were characterized by an average particle size of 226 nm with a polydispersity index of 0.3 and a high loading efficiency. Loaded curcumin showed prolonged release up to 20 h. Furthermore, the obtained formulation showed low toxicity and hemocompatibility in MTT assay. A gellan-based nanohydrogel for piroxicam cutaneous delivery was suggested by Musazzi et al. [113]. They prepared a nanogel with gellan conjugated with cholesterol or riboflavin. The performed skin permeation studies showed that their formulation enhanced piroxicam retention in the epidermis and, at the same time, slowed down the permeation process with respect to the controls. Nanogel can penetrate the stratum corneum, and then gradually disassemble thus diffusing in the viable epidermis reaching the spinosum layer. It represents a novel strategy to target poorly permeable compounds in the dermis which can improve the treatment of cutaneous problems [113].

The microencapsulation process can also be utilized to enhance the viability of probiotic species. Marcial-Coba et al. [114] evaluated the viability of microencapsulated *Akkermansia muciniphila* and *Lactobacillus plantarum.* It turned out that a gellan-based matrix significantly increased the survival of bacterial cells during the simulated stomach passage, enabling future use of this important member of the human colonic microbiota as a probiotic.

### 4.5. Xanthan Gum (XG)

**Figure 11:**
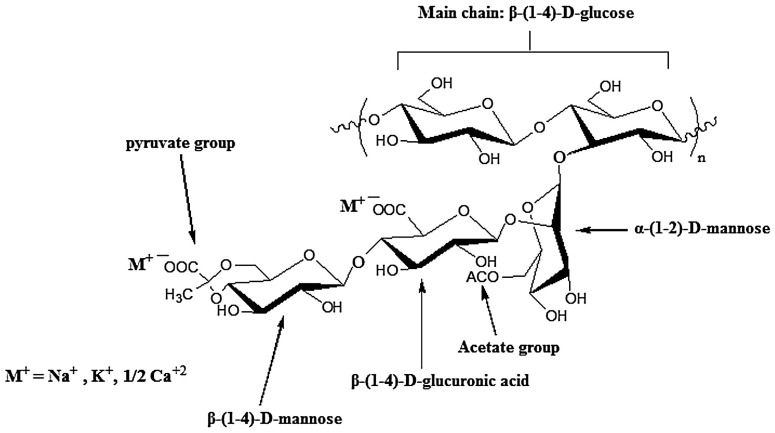
Penta-saccharide repeating unit in XG structure. Reproduced from [117] with permission.

Xanthan gum is a branched polysaccharide biosynthesized by Gram-negative bacteria of *Xanthomonas* genus, most often *X. campestris*. The polymer backbone resembles cellulose, as it is a cellobiose unit (two β-1,4-linked glucose molecules). Every alternating glucose residue, a trisaccharide side chain is attached via α-1,3 bonding. This consists of β-d-mannose (acetylated at position *O*-6)–(1,4)-β-d-glucuronic acid– (1,2)-α-d-mannose (Figure 11). Moreover, apart from the ionizable glucuronate unit, approximately half of the terminal mannose residues are keto-bonded at positions 4 and 6 to pyruvic acid group, with exact contents depending on the fermentation process [115,116]. Its molecular weight falls in the range of 1–50 × 10^6^ g/mol. The polymer hydrates and dissolves well both in cold and hot water. While chain conformation depends on the solution conditions, xanthan gum is regarded as thermally and chemically stable polymer under wide range of pH, ionic strength and temperature, which warrants its application in pharmaceutical, biomedical, food, cosmetic or petroleum industry [115].

Aqueous solutions are highly viscous (even up to millions mPa·s [116]) and display shear-thinning behavior. Above pH 4–4.5, the polysaccharide exists as a polyanion due to the deprotonation of O-acetyl and pyruvate residues, while below this value the groups are in non-ionized state, which results in decreased viscosity. Owing to these properties, xanthan gum is widely used as a thickening or stabilizing agent for suspensions and emulsions. As xanthan gum is non-toxic, biocompatible and biodegradable, it gained FDA approval over fifty years ago (1969). Apart from the abovementioned application of the gum as colloidal stabilizer, XG has found its use mostly in oral and topical drug delivery, in dosage forms such as films or sustained-release matrix (owing to its swelling properties) [118].

Undoubtedly, among the most notable areas of xanthan gum applications is related to its hydrogel generation properties. While monovalent and divalent ions induce a charge-screening effect and reduce XG solution viscosity, trivalent cations act as crosslinking agents. Gelation into elastic network structures may also be triggered by chelating agents [116]. Pure xanthan gels are stable under acidic and neutral pH, as in alkaline conditions ester bonds are hydrolyzed [115]. While there are numerous reports concerning XG hydrogels of various forms, e.g., [119,120], the review below is focused on micro- and nanogel structures and micro-/nanoencapsulation of actives where a crosslinking agent is used. Such formulation technologies have been employed most often for the design of sustained-release oral dosage forms (including gastroretentive ones) or targeted colonic delivery of locally acting drugs. They have also been applied for stability-enhancing encapsulation of herbal extracts or essential oils. An interesting emerging field of xanthan gum hydrogel use is also the potential for tumor targeting delivery systems.

#### 4.5.1. Unmodified Xanthan Gum

Surprisingly, descriptions of unmodified xanthan gum as the single hydrogel former on the micro or nano-scale are rather scarce in scientific literature. A notable exception is the work of Singh et al. on what the authors called ‘trichotomous gastroretentive drug delivery system’ (TGRDDS) [121]. The concept addressed the need to maintain a steady, therapeutic plasma concentration of capecitabine, a drug used in the treatment of colorectal cancer, which is rapidly metabolized to 5-fluorouracil and completely eliminated from systemic circulation within 6 h. In order to overcome this pharmacokinetic hurdle by prolonged capecitabine absorption after oral administration (sustained delivery), a system was designed combining three principles of gastroretention: swelling, mucoadhesion and floating of the dosage form. The former two modes of action were ensured by xanthan gum as the polymer, and floating was realized by incorporation of sodium bicarbonate (with sulfuric acid). XG microspheres with capecitabine and NaHCO_3_ were prepared by water-in-oil emulsion-solvent evaporation method, where the ingredients were emulsified in soybean oil with Span^®^ 80. The droplets were then crosslinked with glutaraldehyde in the presence of small amount of H_2_SO_4_ as acidifier for CO_2_ generation upon hydration of microspheres.

The TGRDDS microparticles were spherical with porous surface, with a mean size between 120 and 243 µm (depending on surfactant concentration). Increasing the proportion of xanthan gum to the drug (from 2:1 to 3.2:1) improved encapsulation efficiency (EE) from 78 to 88% and was related to higher degree of swelling (72–82%) and floating. Unsurprisingly, the increase in NaHCO_3_ level also improved the buoyancy, where up to 91% percent of particles remained unsunken after 12 h in simulated gastric fluid (SGF). The mucoadhesiveness of the designed microparticles was also proven in an ex vivo test on goat intestinal mucosa with texture analyzer (68–88%, increasing with XG content), as well as an in vitro test, where the sample was incubated with rat gut loop, resulting in mucoadhesive retention of 65% of the microparticles. Sustained release from the developed TGRDDS in SGF over 24 h was demonstrated, with a burst release of 20% within the first 30 min through the pores formed by migrating CO_2_, and extended release to 80% within 12 h (in contrast to pure drug, which was completely released from a gelatin capsule in 1 h). Accordingly, in the in vivo test on rat population, the area under the curve (AUC) value was 1.44-fold higher after the administration of the formulation when compared to capecitabine solution, demonstrating the validity of TGRDDS concept [121].

#### 4.5.2. Carboxymethylated Xanthan Gum (CMXG)

Even though trivalent metal ions are known to crosslink xanthan gum as outlined in Section 4.5, in the field of pharmaceutical technology, it has been noted that gelation property of XG in the presence of aluminum cations is weak and it is difficult to produce hydrogel beads from this polymer with simple ionotropic gelation technique, widely applicable to other biopolymers such as alginate [122]. In order to facilitate this, chemical derivatization of xanthan gum has been established, namely carboxymethylation, where O-acetyl group is substituted with carboxymethyl group. This provides more negatively ionizable sites to the polymer chain, which can interact with crosslinking trivalent cations such as Al^3+^ [123]. In this vein, several examples of research have been conducted on hydrogel micro-/nanoparticles of carboxymethylated xanthan gum (CMXG).

Among the earliest examples of such applications is the work of Maiti et al. regarding the encapsulation of bovine serum albumin (BSA) [123,124]. Initially, the authors simply induced gelation by injecting the BSA in sodium CMXG solution into aluminum chloride solution. It was observed that microparticles could be formed only in the pH range of 4–8 and that divalent cations (calcium, barium) were unsuccessful in crosslinking the material. The microparticles shape was found to depend on CMXG concentration (and hence solution viscosity), with optimum spherical shape at 1–2%. Below these values, the microparticles were elongated and flat, while they were elongated with tails above [123]. It was also found that an increase in AlCl_3_ concentration (1–5%) resulted in a larger bead size (as more crosslinked, denser gel hinders water efflux and microparticle shrinkage), lower entrapment efficiency (EE) (drop from 86 to 61%), as well as a less spherical and more indented particle shape [124]. BSA release was pH dependent, with 96–98% released at pH 7.4 and ~40% in simulated gastric fluid (SGF) [123]. Since the authors considered the release in an acidic medium to be excessive for oral protein delivery, the gelation process was modified by adding a coating hydrogel layer to the original microparticles. For this purpose, the microparticles were immersed in sodium CMXG solution and transferred again to AlCl_3_ or alternatively, transferred to calcium chloride solution and immersed in sodium alginate, followed by final alginate gelation in CaCl_2_ solution. The coating procedure reduced BSA release in SGF to 10–15%, as well as slowed the release in PBS. CMXG layer was thicker and retarded the release at pH 7.4 more efficiently than the alginate one, with slightly larger microparticle size (1330 vs. 1290 µm) and final encapsulation efficiency of 79%. The loading process did not affect protein stability [124].

**Figure 12:**
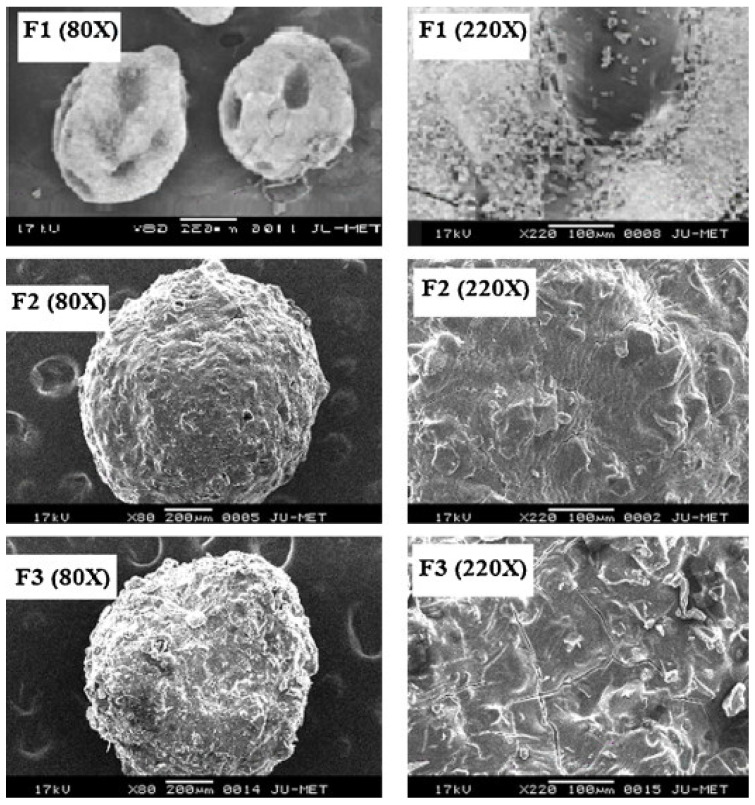
Scanning electron micrographs of resinate-loaded hydrogel beads with 80× and 220× magnification [carboxymethyl xanthan gum hydrogel, prepared with ionic crosslinking (F1), simultaneous ionic and chemical crosslinking (F2) or sequential ionic and chemical crosslinking (F3)]. Reproduced from [127] with permission.

Similar effects of crosslinking conditions were observed in studies on CMXG encapsulation of dilitiazem complexated on an exchange resin [125,126], which was intended to provide sustained release of the hydrophilic drug. Therefore, the findings of the abovementioned work on BSA [123,124] were supported by the results obtained by Ray et al.: CMXG concentration of 1.5–2.5% was necessary for spherical microparticle shape, and aluminum chloride concentration increase had a negative impact on EE. The most pronounced effect on encapsulation efficiency was exerted by gelation time, where extending it from 5 to 20 min led to EE drop from 95% to 79%. Additionally, similar to the work on bovine serum albumin, CMXG microgel alone was unsuccessful in providing sufficient sustained release: the system released approximately 80% of the drug in simulated intestinal fluid (SIF) in 2 h, and experienced even quicker burst to ~30% in SGF within 30 min [125]. To overcome this, diltiazem resinate-loaded CMXG beads were immersed in polyethylene imine (PEI) in order to form polyelectrolyte complex with CMXG, which would reduce pore size and permeability in the hydrogel network. By comparing different PEI concentrations (0.5–2%) and incubation times (5–30 min), it was observed that an increase in both values reduced EE (from 96.5% to 70%) on the one hand, but on the other hand, reduced the drug amount released at pH 1.2 end extended the release at pH 6.8 (with t_50%_ value raised from 90 to 270 min). It was therefore confirmed that improved thickness and tightness of the polyelectrolyte membrane reduced diltiazem migration from the matrix. The optimal formulation (obtained with 1% PEI and 5 min incubation) was evaluated in vivo in rabbits. Compared to diltiazem solution, the developed CMXG-PEI system successfully prolonged the absorption after oral administration and enhanced relative bioavailability to 159% (with c_max_ reduced from 180 to 120 ng/mL and absorption rate constant from 0.854 to 0.625 h^−1^). With the sustained delivery system, a level A in vitro–in vivo correlation (IVIVC) between fraction released and fraction absorbed was also confirmed (R^2^ = 0.96) [126]. In another study of this series on CMXG-encapsulated diltiazem resinate, the effect of different crosslinking methods was explored. Three modes were compared: ionotropic gelation in 2.5% AlCl_3_ for 15 min, simultaneous ionotropic and chemical crosslinking in the solution containing 2.5% AlCl_3_ and 5% glutaraldehyde for 15 min, and sequential mode, where the microparticles were first gelled by 2.5% aluminum chloride, dried and subsequently crosslinked by glutaraldehyde (0.5–5% for 5–30 min). The bead size and morphology were influenced by the choice of method (Figure 12): the Al^3+^-crosslinked microparticles were the largest (1.18 mm), smooth and spherical, but partly collapsed during drying. On the other hand, the combined ionotropic and chemical crosslinking provided spherical microbeads with rough and striated surface (a result of inward shrinkage of the polymer chain during the reaction of aldehyde with –OH groups). The smallest size of 0.85 mm was recorded for the sequentially prepared hydrogel at the highest glutaraldehyde concentration due to more rigid structure. The EE was roughly comparable between the processes (80–88%). In a dynamic in vitro test with pH change, the ionotropically gelled microparticles gave the poorest performance due to looser, less crosslinked structure and free –OH groups available for hydrogen bonding with water, and sequentially crosslinked beads were the most efficient in slowing diltiazem release (mean dissolution time MDT 28.43 s vs. 35.95 at pH 1.2 and 38.73 s vs. 62.66 at pH 6.8, respectively) [127].

An interesting dual application of modified XG was presented by Maiti and Mukherjee in a delivery system intended simultaneously to ensure extended release and improve the solubilization and oral bioavailability of poorly soluble glibenclamide [128]. To realize the latter, C_16_ alkyl chain-grafted-xanthan copolymer was first synthesized and proved to spontaneously organize into micelles in aqueous environment. The drug was then loaded into the polymeric micelles (as a chloroform solution with subsequent solvent evaporation). Finally, the drug-containing micelles were added to sodium CMXG solution, which was next injected to 1% aluminum chloride. For comparison purposes, blank hydrogel microparticles, microparticles with placebo micelles, as well as those containing free glibenclamide without micellar aid were prepared. All the hydrogel particles of approximately 600 µm in size were fairly irregular, with a collapsed and slightly rough surface after drying, regardless whether micelles were incorporated or not. Interestingly, the loading of free glibenclamide resulted in better EE (97%) than the copolymer micelles-encapsulated drug (77%), which was explained by improved solubilization of the drug in micelles and its diffusion out of the hydrogel matrix. In an in vitro release study with a dialysis bag, 82% of glibenclamide was released in 8 h from the micellar dispersion. On the other hand, 22–26% was released from microgel delivery system at the same time, with superimposable profiles and no statistical difference in dissolution efficiency parameters between the microparticles containing micellar and free drug, which was confirmed to be incorporated in non-crystalline form. However, the kinetic Korsmeyer–Peppas modeling revealed different release mechanism: simple diffusion for the free drug hydrogel, and complex non-Fickian for the micellar hydrogel. Finally, in vivo activity of the formulations was compared in rabbits. The micellar dispersion displayed the highest level of blood glucose reduction 5 h after the oral administration (57%), followed by a decline below 30% after 8 h. On the other hand, the % of glucose reduction after the administration of extended release microparticles raised steadily and continuously up to 35–37% at 8 h, without significant differences between the micellar and free glibenclamide hydrogels [128].

**Figure 13:**
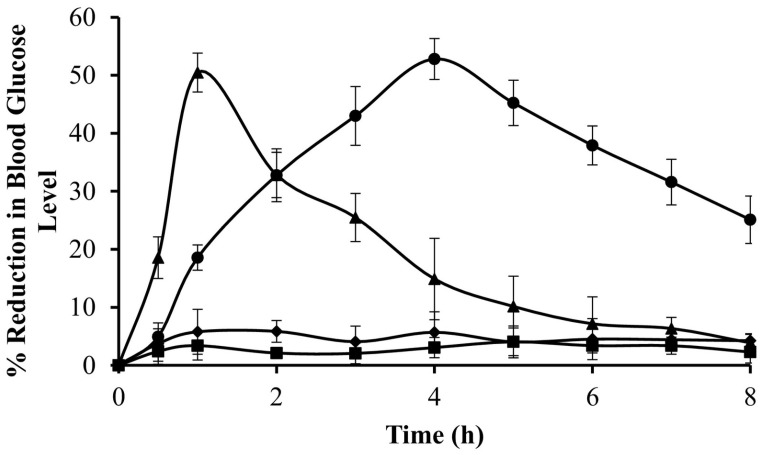
Preclinical efficacy of XGD6 hydrogel particles [CMXG + CEXG acetylated hydrogel containing repaglinide]. Key: diabetic control (◆), negative control (■), pure repaglinide suspension (▴) and XGD6 formulation (●). Reprinted from [122] with permission.

Recently, another XG derivative has been tested for microgels in drug delivery: carboxyethyl xanthan gum (CEXG). The study aimed to design an extended release system of another antidiabetic drug, repaglinide, and stabilize the variations in its plasma concentrations and blood glucose levels [122]. CMXG and CEXG were synthetized and mixed at different proportions; the drug-containing solution was then crosslinked by AlCl_3_. Moreover, after drying of the collected hydrogel particles selected formulations were further acetylated with acetic anhydride for surface hydrophobization. It was found that CEXG alone produced hydrogel with insufficient strength, which formed flattened disks after drying. When mixed with CMXG, the resulting microparticles were generally spherical. The incorporation of repaglinide reduced the folding and collapse of the surface, while acetylation provided roughness. In terms of size (839–1176 µm), acetylated microbeads were smaller than non-modified ones, and increase in CEXG content increased the solution viscosity, which contributed to microparticles enlargement. The encapsulation efficiency (EE; 43–92%) was affected both by the hydrogel composition and the acetylation. High amounts of CEXG were detrimental in this aspect, as highly viscous polymer with weak gelling capacity produced inhomogeneous dispersion, from which the drug was prone to leaching. On the other hand, at the proportion of 33.3%, the addition of CEXG improved the encapsulation efficiency when compared to pure CMXG. Simultaneously, acetylation of the hydrogel was not beneficial for EE of this particular drug, since upon the reaction of acetic anhydride with the hydrogel moisture, acetic acid was formed. As repaglinide is well soluble in acidic environment, the API tended to leach. Similarly, both of the studied formulation factors affected the swelling and in vitro release in a dynamic test. Lowering CEXG contents reduced the swelling and slowed down the release rate. However, only the highly hydrophobic, low-swelling network of acetylated hydrogel enabled the complete release of the drug in 8 h instead of 4 h. The acetylated microgel was demonstrated to effectively stabilize the blood lowering activity of repaglinide for 8 h despite later onset when compared to pure drug in suspension (Figure 13) [122].

#### 4.5.3. Xanthan Gum Grafted Copolymers

In the area of micro- and nanogels for drug delivery, some research has been performed on grafted copolymers based on xanthan gum. Among such examples is the work on XG copolymerization with glicydyl methacrylate (GMA) as a potential drug carrier which would combine chemical stability and pH resistance of synthetic polymers with bioadhesiveness and biocompatibility of natural gums. Simultaneous copolymerization, crosslinking with different methacrylic monomers, as well as theophylline loading took place in a single reaction. The resulting microparticles were spherical, of porous surface and cross-section. Their properties depended on the applied methacrylic crosslinker: longer chains increased surface roughness and decreased hydrophilicity. Drug release from the microparticles based on XG copolymer was slower than from those made only from GMA and several kinetic models were a reasonably good fit [129].

An interesting and in-depth investigation was carried out on XG copolymeric nanogels for controlled, targeted release of doxorubicin. Two such delivery systems have been described so far, taking advantage of the principle that internal environment of cancer cells is more acidic (pH 5–6) and has a higher glutathione (GSH) concentration than blood and normal cells [130,131]. By fine-tuning the release properties of nanoparticles to respond to such conditions, targeted delivery of the cytotoxic drug to tumor could potentially be achieved without affecting healthy cells. According to this, Zhang et al. prepared nanogel particles in a single, one-pot reaction of grafting and crosslinking XG with previously prepared cystamine tetra-acylhydrazine (CTA); in such a way, amide bonding formed between amino groups of the crosslinker and carboxyl groups of the gum. Subsequently, doxorubicin was chemically loaded into blank nanoparticles by nitrile bonding to a acylhydrazide moiety. The nanoparticles were elliptical in shape and their size decreased from 510 to 218 nm with an increase in the XG:CTA proportion (from 1:0.0183 to 1:1.127) due to a denser nanogel structure and more hydrophobic interactions [130]. In a second study, Feng et al. grafted 2-acrylamido-2-methylpropane sulfonic acid (AMPS) onto XG in a single one pot reaction in the presence of newly synthetized crosslinker *N*,*N*-bis(acryloyl) cystamine (BACy). Subsequently, doxorubicin was incorporated into the nanoparticles by ionic interaction with sulfonic groups of the copolymer. Increasing the proportion of AMPS to XG from 2.5:1 to 10:1 and consequently, increasing grafting rate, slightly enlarged nanoparticles’ size from 110 to 180 nm (due to stronger hydration properties of the nanogel with more sulfonic groups). Moreover, it also increased the drug load from 8.6 to 15.2% owing to higher degree of doxorubicin chemical bonding [131].

For both of the described delivery systems the desired pH- and redox-responsive release was demonstrated. The cumulative amount released at pH 5.0 (corresponding to cancer cells) vs. at pH 7.4 (physiological value) was 29.9% vs. 44.9% and 21.1% vs. 38.6% for XG-CTA and XG-AMPS, respectively, either due to the destruction of acylhydrazone bonds or protonation of sulfonic groups in acidic environment. When glutathione as reducing agent was added to the medium to represent the redox capabilities of cancer cells, the release of doxorubicin was improved even further to 72.1% (XG-CTA) and 63.5% (XG-AMPS). As disulfide bridges were broken, some of the crosslinked nanogel structure was destroyed, which was also evident from the swelling and size increase in the nanoparticles. The authors also verified that blank nanogel did not reduce 3T3 cells viability [130,131], but doxorubicin-loaded XG-AMPS nanoparticles were indeed cytotoxic towards HeLa cancer line, subjected to uptake most likely by endocytosis [131].

Another example of xanthan-gum grafted systems is the work on hydrogel beads for delayed, extended release of ketoprofen for enteric delivery to reduce the drug’s irritative properties [132,133]. Kulkarni and Sa grafted polyacrylamide (PAAm) onto XG and subjected the copolymer to alkaline hydrolysis to convert amide groups into carboxyl ones for acid resistance. Ketoprofen was then dispersed in the copolymer solution, which was added to an aluminum chloride gelling bath (20–40% *w*/*v*). The resulting hydrogel beads were spherical with a rough surface, and their size decreased from 1003 to 960 µm with an increase in gelling agent concentration. A higher concentration of AlCl_3_ also improved EE (76–86%) due to leakage prevention by a denser network. Compared to microparticles made of pure XG, the XG-PAAm hydrogel not only prevented drug release at pH 1.2, but also slowed down the release in PBS (complete release in 8 vs. 5 h), confirming both enteric and prolonged delivery. The release rate was affected in a contradictory way by ionic crosslinker concentration and drug load—higher amounts of AlCl_3_ strengthened the hydrogel structure, while ketoprofen weakened it. The prepared delivery system was tested in vivo on rats—delayed anti-inflammatory and analgesic activity was observed based on the reduced injury edema and reaction time against a pain stimulus. The histopathological evaluation proved the intended gastroprotective properties of the designed microparticles: administration of pure ketoprofen induced ulceration, bleeding and even necrosis of gastric mucosa. On the other hand, when the drug was administered in hydrogel microparticles, only minor congestion and erosion were observed [132]. In a follow-up study, the authors aimed to improve the beads’ entrapment efficiency and mechanical strength by ionotropic gelation of interpenetrating polymer network (IPN) of XG-PAAm with carboxymethyl cellulose. While the microparticle size (910–1081 µm) and pH-dependent swelling and release remained similar, the EE of amorphous ketoprofen was enhanced to 82–93% [133].

**Figure 14:**
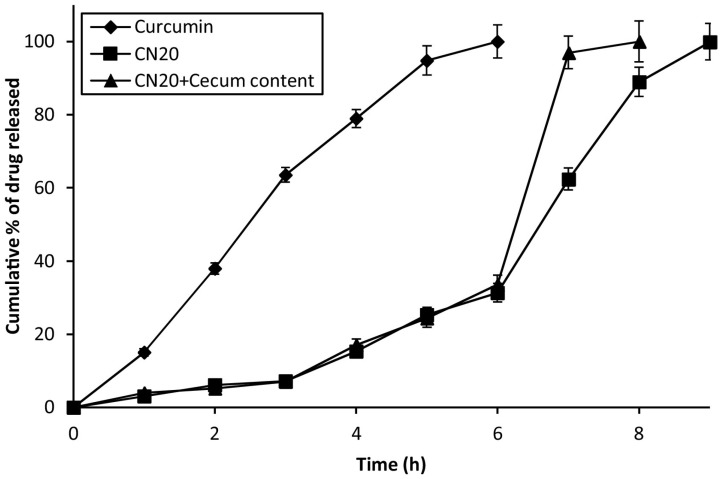
Dissolution profile of pure drug [curcumin] and CN20 NPs [curcumin-loaded polyacrylamide-grafted-xanthan gum hydrogel]. Reproduced from [134] with permission.

An interesting application of the same grafted copolymer XG-PAAm was presented by Mutalik et al. [134]. The delivery system for curcumin was designed with the aim to simultaneously improve its bioavailability and provide targeted colonic delivery for inflammatory bowel diseases, taking advantage of the combination of pH-dependent degradation of polyacrylamide and biodegradation of xanthan gum by gut microbiota. Several preparation factors were studied: curcumin and copolymer concentrations, phase volumes and sonication parameters during the emulsification of curcumin DCM solution into aqueous XG-PAAm solution, AlCl_3_ concentration in ionotropic gelation. The optimal nanoparticles were smooth and spherical, with a mean size of 425 nm and EE 15–17.5%. Saturation solubility of curcumin loaded into the nanogel was increased 26–48 times, depending on the medium, which may be attributed to at least partial drug amorphization. Compared to pure curcumin, the designed delivery system successfully slowed the release at pH 1.2 and 4.5, while the release was fast after the media change to simulated colonic environment (pH 7.2 and 6.8) in a dynamic, sequential in vitro test. Moreover, the study validated the concept of applying xanthan gum for controlled release relying on microbial biodegradation—the addition of 1% rat cecal content to the dissolution medium quickened the release when compared to pure buffers of pH 7.2 and 6.8 (Figure 14). The developed formulation was tested in vivo in a rat colitis model against pure curcumin and sulfasalazine as standard treatment. The drug’s systemic availability from the nanoparticles was increased, with 3-fold and 2.5-fold higher values of c_max_ and AUC, respectively. Preclinical efficacy in colitis was also demonstrated: a drop in body weight was prevented and normal colon length was restored to a similar degree as after the administration of sulfasalazine, and to a higher extent than after pure curcumin. In terms of levels of inflammatory markers (myeloperoxidase and tissue nitrite), the curcumin-loaded XG-PAAm nanogel performed even better than standard sulfasalazine [134].

#### 4.5.4. Combinations of Xanthan Gum with Other Natural Gums

Apart from micro- and nanogels based on xanthan gum and its modifications alone, several studies have been carried out on its combinations with other polymers: polysaccharides (including other types of gums) and other natural or synthetic polymers.

**Figure 15:**
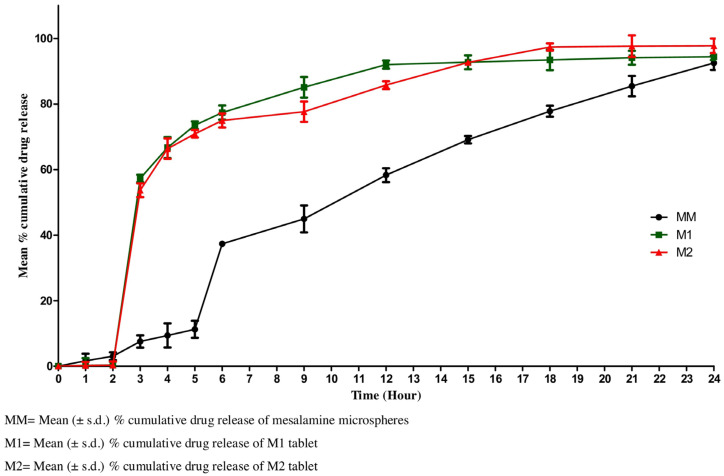
Mean (±s.d.) percentage cumulative release of mesalamine microspheres [xanthan gum and guar gum hydrogel] and its marketed preparations in the presence of 4% *w*/*v* rat cecal contents. Reproduced from [135] with permission.

Based on an assumption similar to the referred above work of Mutalik et al. [134], a colon-targeted delivery system of mesalamine for the treatment of ulcerative colitis was developed. The combination of xanthan and guar gum served both as the controlled release carrier subjected to biodegradation by the gut microbiome and as a prebiotic in order to support the survival of symbiotic bacteria in contact with mesalamine. Since the drug may negatively impact their viability, not mitigating this effect may hamper further release driven by matrix biodegradation [135]. With this rationale, Kaur et al. prepared mesalamine-loaded microspheres by first emulsifying aqueous guar:XG (1:0.5) solution with API into external oil phase; the emulsion droplets were crosslinked with glutaraldehyde. The obtained microspheres were smooth, with a d90 value of particle size distribution of 291 µm and EE of 69.25%. Moreover, their processability was also favorable, with good flow properties and compressibility. The formulation was compared against two commercial mesalamine matrix tablets in a dynamic in vitro release test with sequential media change: pH 1.2 for 2 h, PBS pH 6.8 for 3 h, followed by PBS pH 6.8 with 4% rat cecal content under anaerobic conditions up to 24 h. Whereas the marketed products released the majority (over 60%) of mesalamine soon after the switch from SGF to SIF; at a similar time point, the microspheres released only approximately 10% of the drug. Instead, upon changing to simulated colonic conditions, the designed formulation started a steady, controlled release of mesalamine, up to 80% over the course of the next hours (Figure 15). This behavior confirmed the mechanism of bacterial degradation of polysaccharide matrix. To further support the findings, the microspheres were compared in vivo on a rat colitis model against other treatment options: pure mesalamine, probiotics administration, mesalamine + probiotics, the developed microspheres incorporating mesalamine, and microspheres + probiotics. Based on the stool and body weight observation, as well as histopathological evaluation, the recovery was the fastest in the group which received the combined treatment of XG:guar gum microspheres incorporating mesalamine and probiotics. What is more, to further support the findings of in vitro release test, the authors collected cecal content from the different treatment groups and demonstrated that drug release rate in simulated colonic environment was varied depending on the composition and condition of gut microbiota, related to the received treatment. In conclusion, the study proved the validity of the coadministration of probiotics with prebiotic polysaccharide-based targeted colonic drug delivery system, as well as their advantage over synthetic, pH sensitive matrices [135].

Another example of a gum applied in combinations with XG for micro- or nanoencapsulation is locust bean gum (LBG). In a study on biodegradable microparticles for extended release of celecoxib, the gums’ solution with the drug was emulsified in paraffin as external phase and crosslinked with glutaraldehyde [136]. Emulsification conditions were optimized and a Central Composite Design was applied to study the effects of drug: LBG proportion and its ratio to XG, which were found to be non-linear with respect to EE (58–96%) and drug load (19–38%), in general enhancing these values with an increase in polymers concentration. The resulting microparticles of mean 196 µm were spherical with smooth, locally collapsed surface; their friability was assessed as good and flow properties as reasonably good. The extended release behavior was comparable to commercial Celedol tablet, and an increased proportion of LBG was observed to slow down the release rate [136].

**Figure 16:**
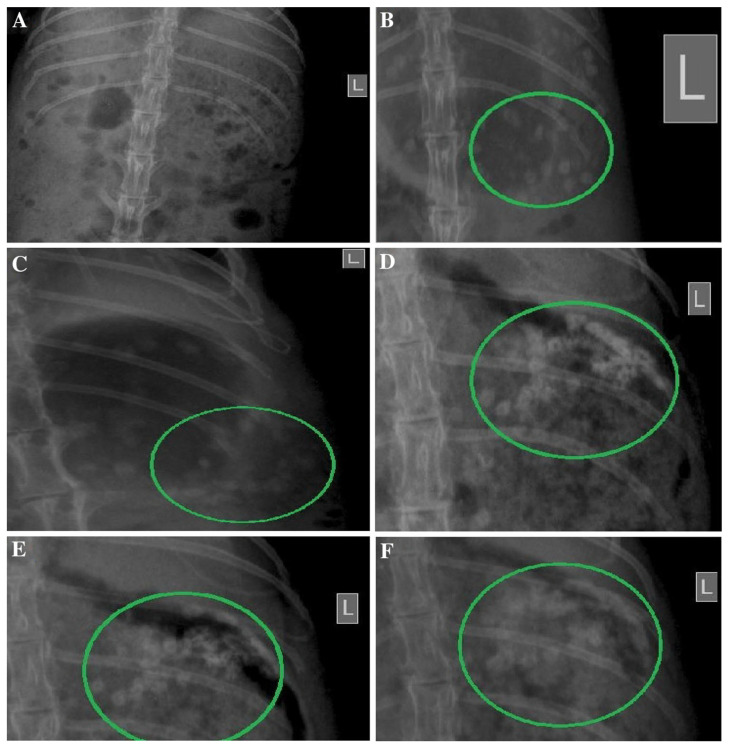
Radiographic images taken on the rabbit during radiological studies [gastric retention of pectin-xanthan gum-locust bean gum hollow/porous floating beads as pulsatile drug delivery system] at predetermined time intervals (**A**–**F**). The presence of the beads was marked with the green circles. Reproduced from [137] with permission.

A more noteworthy work employing these two natural gums was presented by Taranalli et al., who developed hollow/porous floating beads for pulsatile drug delivery of metoprolol [137]. The gastroretentive delivery system was intended to extend the lag phase followed by burst release. Interestingly, the biopolymeric basis for the beads was the combination of pectin and XG, and screening studies were performed to select the third polymer, based on encapsulation efficiency and release. Although carboxymethyl cellulose, guar and gellan gum were tested, locust bean gum was chosen for the optimized formulation. The metoprolol-containing solution of XG, LBG and pectin with added sodium bicarbonate (floating agent) was crosslinked by dropwise addition to calcium chloride solution. Regardless of the biopolymers proportion, the beads (1.134–1.198 mm, EE 54–77%) were spherical with hollow core and porous surface due to CO_2_ formation. During the formulation screening study, it was observed that good buoyancy (expected to last for 24 h) was correlated with high porosity and low bulk density. In a sequential in vitro release test, the intended behavior of the delivery system was confirmed: lag phase at pH 1.2 with 6–14% released in 6 h, followed by burst metoprolol release after pH change to 6.8 according to first order kinetics. This was provided by the combination of calcium pectinate and LBG in the matrix, which are insoluble in acidic media. Finally, the gastroretentive properties of the developed drug delivery system were confirmed in vivo by an X-ray study on rabbits—the hollow/porous beads remained clumped in the stomach for 8 h (Figure 16) [137].

**Figure 17:**
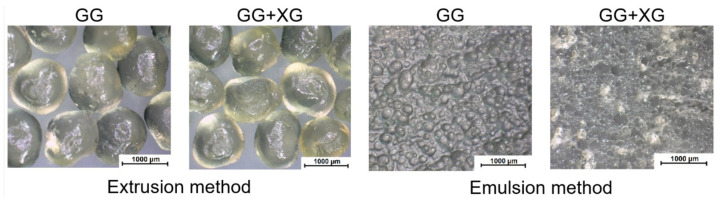
Microscope images of swollen microspheres prepared from gellan gum (GG) and gellan gum with xanthan gum (XG) by the extrusion and emulsion methods. Scale bar = 1000 µm. Reproduced from [138].

Among xanthan combinations with other natural gums, gellan has also been used in a work on the encapsulation of *Calendula officinalis* herbal extract, although the authors indicate an intended cosmetic application rather than drug delivery [138]. However, the study might be of interest to pharmaceutical scientists from a technological point of view as well. Kozlowska et al. compared two methods of microspheres made of pure gellan gum or gellan + xanthan gum. The polymers solution (1.5% GG or 2.5% GG + 1.5% XG) was either directly extruded to CaCl_2_ solution, or first emulsified in paraffin by Tween^®^ with subsequent addition of calcium chloride. The preparation method had a significant impact on the size of microspheres (Figure 17): the crosslinked emulsion droplets were nearly 100 times smaller than solution-derived microparticles (6–7 µm vs. 384–448 µm). The addition of XG increased the size when compared to gellan gum alone, but on the other hand improved encapsulation efficiency. The emulsion method also performed better in terms of EE than extrusion method, with the highest EE value for the herbal extract at ~40%. The release behavior of the microspheres was tested in a pharmaceutically irrelevant setup, but it was revealed that smaller, emulsion-derived microparticles enabled faster release. Regarding polymeric composition, the addition of XG was observed to slow down the release. Moreover, the prepared *Calendula officinalis* microspheres were further incorporated into matrices composed of collagen, gelatin and hydroxyethylcellulose, prepared by freeze drying and chemical crosslinking (with 1-ethyl-3(3-dimethylaminopropyl) carbodiimide and N-hydroxysuccinimide). Compared to blank matrices, microsphere loading decreased their swelling and porosity, as well as modified their mechanical properties and degradation rate to a degree depending on the composition and preparation method of the microspheres [138].

#### 4.5.5. Combinations of Xanthan Gum with Other Polymers

Apart from combining xanthan with other natural gums for micro-/nanogels, XG or its derivatives has been used with popular polysaccharides. Among these is carboxymethyl cellulose (CMC) used in an interpenetrating polymer network (IPN) system with CMXG, crosslinked ionotropically with aluminum chloride in the presence of Tween^®^ in a water-in-water emulsion preparation method. The obtained hydrogel beads were spherical with the size between 1.08 and 1.42 mm, depending on AlCl_3_ concentration (crosslinking density). Increasing the CMC:CMXG ratio from 1:1 to 2:1 decreased entrapment efficiency of diclofenac from 97 to 77%, as better soluble cellulosic polymer enabled drug leaching. Extended release was confirmed, with the rate dependent on crosslinking density as per gelling agent concentration and time [139]. Another example of IPN hydrogel beads was prepared by the extrusion of XG and sodium alginate solution into calcium chloride gelling bath. An increase in xanthan gum amount and solution viscosity resulted in the bead size increase (from 1.31 to 1.54 mm), more regular and indented shape, but also improved EE (from 71 to 81%), as well as enhanced the swelling and prolonged the release rate of glipizide [140].

Chitosan, a positively charged polysaccharide, has also been used as a polyelectrolyte complex with polyanionic xanthan gum for the crosslinking encapsulation of essential oils as potential antibacterial agents, with the aim to improve their stability [141,142]. Skalickova et al. tested combinations of chitosan with several polysaccharides, including gums (XG, guar, alginate, and pectin), for the nanoencapsulation of essential oils (*Thymus vulgaris*, *Rosmarinus officinalis* and *Syzygium aromaticum*) for potential prevention of diarrheal diseases in monogstric farm animals. Oils emulsions in polymer solutions were crosslinked ionotropically with tripolyphosphate and the smallest nanocapsules were obtained from chitosan alone (100 nm), while the largest from its combination with alginate (500 nm), with chitosan-XG falling in the middle. EE was in the range of 40–60%, depending on essential oils and gelling agent concentration. The release was tested in a non-compendial setting and in general, different essential oil ingredients were released in a different way depending both on the medium pH and formulation, but burst release in 3 h followed by an extended phase was observed. However, nanoencapsulation decreased the antibacterial activity compared to pure essential oils [141]. A somehow more promising result was achieved by Li et al. in a study on cinnamon essential oil encapsulation process based on a Pickering emulsion template. First, the emulsion with the oil as internal phase, stabilized by silica was prepared in chitosan aqueous solution. Then, coacervation was induced by electrostatic interaction with XG and finally, the crosslinking was performed with glutaraldehyde. The microcapsules (5–10 µm) were spherical and smooth, without indentations, with EE of 50%. Thermogravimetric analysis confirmed the improved thermal stability of the encapsulated cinnamon essential oil. The formulation displayed antibacterial activity against *S. aureus* and *E. coli*, although no comparison with pure essential oil was made [142].

Finally, non-polysaccharide polymers have also been used in xanthan gum hydrogels for drug microencapsulation. Ray et al. designed IPN of XG and polyvinyl alcohol (PVA) by emulsifying the diclofenac-containing solution into paraffin by Span^®^ 80, and further crosslinking with glutaraldehyde [143]. The microspheres were smooth and spherical, and their size in the range of 310–477 µm shrunk with increasing glutaraldehyde concentration due to more rigid crosslinking, while an increased proportion of XG provided larger emulsion droplets and higher viscosity, translating into larger microparticles. EE (61–84%) was affected in a contradictory way by crosslinker and XG concentration, as the presence of xanthan gum produced more loose matrix structure (lower encapsulation efficiency), whereas higher degree of crosslinking improved the EE value. The same effects relating to hydrogel density were also observed for the swelling and for diclofenac release rate: higher contents of XG favored water uptake and faster release. The prolonged drug delivery from XG-PVA IPN microspheres was corroborated by a level A in vitro–in vivo correlation between fraction released and fraction absorbed in an pharmacokinetic study on rabbits (R^2^ = 0.99). Compared to diclofenac solution, the formulation provided both the delayed t_max_ (2 vs. 4 h) and improved relative bioavailability (169%) [143].

**Figure 18:**
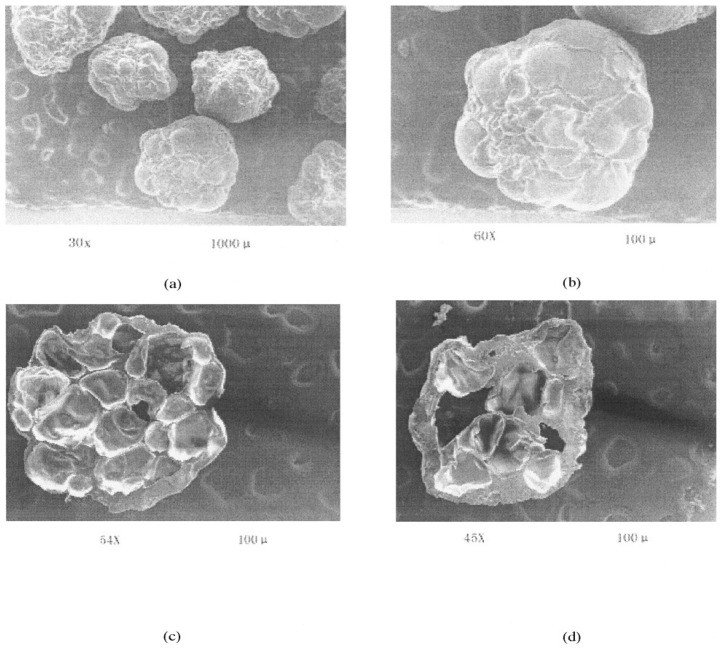
Schematic SEM micrographs of blank microspheres with microballoons inside: (**a**,**b**) photographs of FI [gelatin:xanthan gum 1:1], (**c**) cross-section photograph of FI, and (**d**) cross-section photograph of FIII [gelatin:xanthan gum 3:1]. Reproduced from [144] with permissions.

Among the considered drug delivery systems, one example has also been found where xanthan gum was employed with gelatin for the development of gastroretentive, floating microspheres encapsulating theophylline as a model drug by Yang et al. [144]. Emulsion of the gelatin-XG solution was prepared with paraffin as external phase (without an additional emulsifier) and crosslinked with glutaraldehyde. The resulting hydrogel particles may be seen as quite unique in their morphology as what the authors termed as ‘microspheres with microballoons’. The particles of several hundred microns themselves were spherical, but with several blister-like structures (Figure 18). This was caused by air entrapment during the gelation process and its further expansion without the way of escape. In addition to porous microsphere intersection, this feature contributed to the buoyancy of the system. Formulations with higher XG contents were characterized by larger microsphere size with more ‘microballoon’ structures with thinner walls. This was related to higher solution viscosity on the one hand, and better emulsifying capability on the other hand, resulting in more entrapped air and looser structure. The microspheres were also subjected to 24 h buoyancy test and it was observed that samples with higher gelatin contents sank earlier, corroborating the beneficial effect of XG gum for the floating capability of more porous microspheres. What is more, in correlation to increased microparticles size, a higher proportion of XG to gelatin also improved the EE (56 to 85% with an increase from 1:7.5 to 1:1). Although different formulations gave varied performance in terms of buoyancy, the drug release in a pH 1.2 medium was comparable, with 90% achieved in 600 min of the in vitro test [144].

### 4.6. Tracaganth

Tragacanth is an exudate gum derived from over 2000 of different plant species belonging to *Astragalus* genus [145]. The polysaccharide components of the exudate display molecular mass up to 850 kDa. It is noteworthy that the exact composition of the natural product collected from different regions and different plant species may reveal significant variations regarding monosaccharide moieties, methoxylation degree and the ratio between soluble and insoluble compounds. The common feature of gums derived from different sources was the presence of arabinose, glucose, xylose, galactose, rhamnose, fucose and galacturonic acid moieties. However, the proportions of the particular components, as well as physicochemical properties of the final product, may be different [146]. The main polysaccharide components are water-insoluble bassorin (60–70%) and water-soluble tragacanthin (30–40%) [147]. The first one swells and forms a gel upon contact with the other, while the other one dissolves and forms a colloidal sol. The mentioned fractions are also composed of different polysaccharides—for example tragacanthin consists of ethanol-soluble arabinogalactan and insoluble tragacanthic acid containing galacturonic acid units in its backbone, and other monosaccharide moieties (galactose, fucose and xylose) in the side chains [148]. According to Boamah et al. [145], the exact chemical structure of insoluble bassorin remains unknown. However, tragacanth is recognized as anionic, highly branched polysaccharide and these structural features are important in terms of polymer properties and applications. Tragacanth, similarly to other natural gums, is utilized in many industrial and scientific areas, including food manufacturing, wastewater treatment and construction industry [145]. Due to its excellent thickening properties, biocompatibility and non-toxicity, the polysaccharide can also be used as an excipient in various types of dosage forms [149,150,151], tissue engineering [152] and wound healing [153,154]. Moreover, it can decrease interfacial tension and can be used as a stabilizer in emulsions and suspensions [155]. Numerous studies available in the literature present also micro- and nanogels prepared with the use of native or chemically modified tragacanth.

Rahmani et al. [156] investigated spherical microgels obtained with the use of water-soluble fraction of tragacanth and graphene oxide. As a crosslinking agent, Ca^2+^ and Ba^2+^ cations were used, while calcium carbonate particles were applied as a porogen. The particles were investigated as a potential carrier for rivastigmine, cholinesterase inhibitor accepted in Alzheimer’s disease treatment, in this study utilized as a model drug. The obtained beads were tested for cytotoxicity, swelling behavior and in vitro drug release at pH = 1.2 and 7.4. The drug was released in pH-dependent manner, which was most probably related to ionization of carboxylic groups of the polymer and graphene oxide. The drug release behavior was correlated with swelling. Higher amounts of the drug were released in simulated intestinal fluid. The microparticle matrix showed no cytotoxicity towards human fibroblasts below the concentration of 125 μg/mL.

Veeramachineni et al. [157] prepared carboxymethyl tragacanth-based microparticles with diclofenac sodium as a model drug. The analyzed system was manufactured by ionotropic gelation with aluminum chloride. The microbeads showed minimal cytotoxicity and pH-responsive swelling behavior, with the maximum swelling degree at pH = 7.0. The observed phenomenon was related to the ionization of carboxylic groups and repulsive interaction between them. Another factor affecting the swelling ratio was the amount of the crosslinking agent.

**Figure 19:**
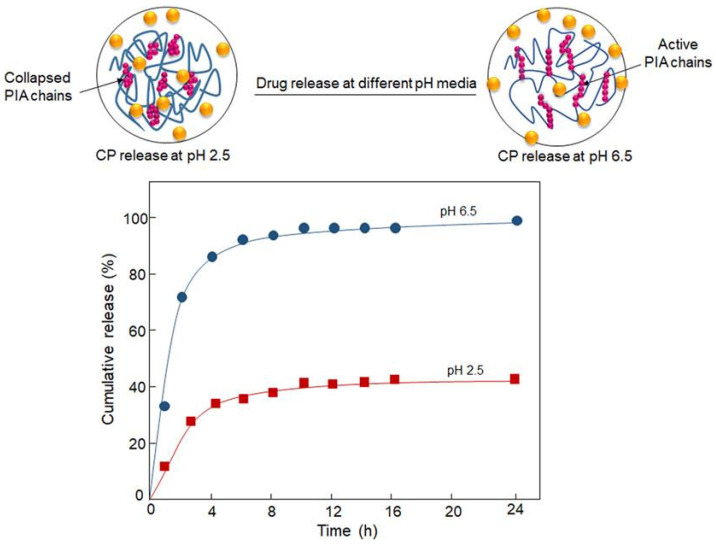
In vitro cisplatin release from nanoparticles at pH = 2.5 and 6.5. Reproduced from [158] with permission.

Verma et al. [158] described tragacanth-based smart nanogels for the delivery of cisplatin, widely applied anticancer drug. Natural gum was grafted with poly(itaconic acid) in order to provide better pH-sensitivity of the product. Nanoparticles coated with lecithin layer were prepared from oil-in-water nanoemulsion by antisolvent precipitation. The particles displayed polygonal shape and their diameter did not exceed 100 nm. The drug release studies performed at pH = 2.5 and 6.5 confirmed that the obtained formulation was pH sensitive, as significantly higher amount of the drug was released at higher pH (Figure 19). The observed effect was related to the swelling behavior of the polymer. At lower pH its carboxylic groups remained at a non-ionized state which prevented swelling and allowed only for partial drug release. The cytotoxicity studies revealed non-toxic character of the unloaded polymer carrier and dose-dependent cytotoxicity of cisplatin-loaded nanoparticles toward cancer cell lines.

Another example of tragacanth-based nanoparticles in drug delivery was presented by Sadrjavadi et al. [159]. In the study, a de-esterified form of tragacanth was used along with chitosan in a polyelectrolyte complex. The de-esterification procedure was applied to improve water solubility and the ability of the polymer to interact with oppositely charged molecules, such as chitosan or metal cations. The aim of the study was to improve some unfavorable pharmacokinetic features of methotraxate, including short plasma half-life, as well as poor solubility and permeability. The particle diameter depended mostly on the concentration of tragacanth derivative, while the zeta potential and the polydispersity index were affected mainly by chitosan molecular weight. The drug was released in a sustained way and 57% of the incorporated drug was released within 9 days of the study. However, the cytotoxicity assay performed with the use of human breast cancer and human colon adenocarcinoma cell lines revealed the investigated formulation performed worse than unloaded methotrexate. The carrier alone did not show any signs of cytotoxicity, which is promising in terms of its potential application. Moreover, the uptake of the nanoparticles was confirmed in the case of breast cancer cell line as a result of active endocytosis.

### 4.7. Carrageenans

**Figure 20:**
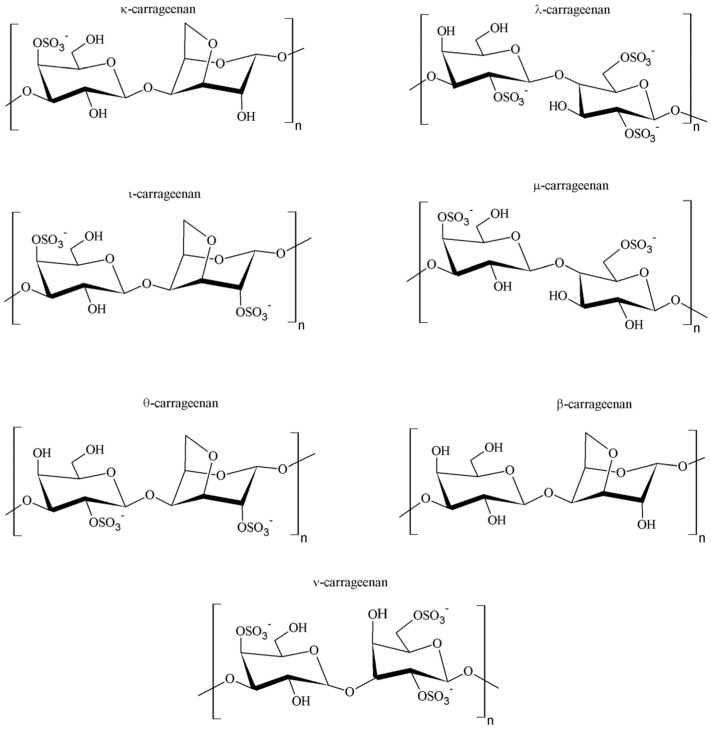
Carrageenan structures. Linear chains of repeating galactose units in D configuration and 3,6-anhydrogalactose copolymer, joined by alternating α-1,3 and β-1,4 glycosidic linkages. Reproduced from [160].

Carrageenans are polysaccharides produced by red algae belonging to *Rhodophyta* class. Structurally, they are anionic galactans, with the main backbone consisting of repeating D-galactose and 3,6-anhydrogalactose moieties connected with alternating α-1,3 and β-1,4 glycosidic bonds. The anionic character of the polymers is related to the presence of sulfate groups. Considering the sulfate content, the polymer solubility and source of origin, carrageenans can be divided into a few groups, including κ-, λ-, ι-, μ-, θ- and β-carrageenan (Figure 20) [160,161].

**Figure 21:**
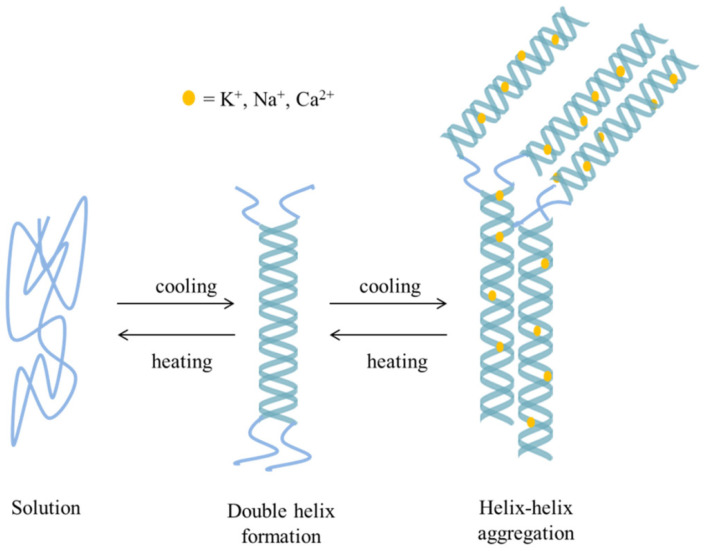
Scheme of carrageenan gel formation. The structure of κ- and ι-carrageenan allows segments of the two molecules to form the so-called double helices which bind the chain molecules in a three-dimension network. The associated counterions such as Na^+^, K^+^ and Ca^2+^, are also required to induce the sol-gel transition of the referred types of carrageenan. Reproduced from [160].

Taking into consideration industrial applications of different carrageenan types, κ, λ and ι are the most important and also the most frequently investigated ones. The main difference between these three forms is the number of sulfate groups in the repeating disaccharide unit. κ-Carrageenan is characterized by the presence of one sulfate substituent, while ι and λ forms have two and three groups, respectively [161]. As strongly hydrophilic compounds, carrageenans dissolve in water and are insoluble in organic solvents. Depending on the structure, particular forms may differ significantly in terms of solubility in various conditions. For example, λ-carrageenan displaying high content of polar sulfate groups and no 3,6-anhydrogalactose moieties revealing low polarity, is highly water-soluble. The solubility parameter depends also on the salt form. Usually, potassium salts reveal worse solubility than sodium salts. Moreover, the available carrageenan forms differ in terms of gelation ability, as λ-carrageenan does not undergo a gelation process and forms only polyelectrolyte solutions. This effect is related to structural differences between three mentioned forms and their gelation mechanism (Figure 21). The structure of λ-carrageenan does not allow for double helix formation; therefore, the transition to gel is not possible.

Carrageenans are frequently utilized in the food industry as thickeners, stabilizers and emulsifying agents [162]. Similarly to other polysaccharide gums, they can also be applied as gelling agents and binders in pharmaceutical technology [161]. On the other hand, carrageenans also display their own biological activities, including antiviral, antibacterial, and antihyperlipidemic activities [163]. They are also well recognized and widely applied in experimental procedures involving inflammation, as they are used to induce inflammatory processes in laboratory animals [164]. Biological activities of carrageenans are also a source of some toxicity-related concerns. The compounds can be safely administered orally, either as food additives or excipients in oral dosage forms. However, the presence of sulfate groups in carrageenan molecule can be related to immunological and blood coagulation side effects after parenteral administration [165,166]. As was already indicated by other authors [167], special attention should be paid to safety whenever parenteral dosage forms or biomaterials intended for contact with blood are taken into consideration. Currently, the role of carrageenan in inflammatory bowel diseases is widely discussed [168,169,170] in context of its pro-inflammatory properties and the results of some recent studies [171,172,173]. So far, no clear conclusions have been made and carrageenans are still extensively investigated as potential excipients in drug delivery, including also micro- and nanogels.

Sarıyer et al. [174] presented hydrogel beads prepared with the use of alginate and κ-carrageenan forming double network. The obtained microgels were pH sensitive and the aim of the study was to design novel efficient dosage form for protein delivery. As a model active compound, bovine serum albumin (BSA) was applied. In this formulation, alginate was used to improve the mechanical properties of the polymer matrix, while carrageenan was supposed to extend BSA release in simulated intestinal fluid. The beads were formed by ionotropic gelation with a mixture of calcium and potassium chlorides as crosslinking agents. The highest encapsulation efficiency was obtained at pH below the isoelectric point of albumin. The release study was performed at simulated gastric fluid for the first 2 h and then the dissolution medium was exchanged for simulated intestinal fluid. It is noteworthy that only approximately 10% of albumin was released in the first stage of the experiment, which is promising in terms of controlled drug delivery.

Arunagiri et al. [175] prepared microparticles with the use of κ-carrageenan, gelatin and tannic acid. The described formulation was designed as a potential hemostatic agent. The particles were prepared with polyelectrolyte complexation, as both polymers are oppositely charged and can interact with each other. The obtained formulation was tested for swelling behavior, cytotoxicity and hemocompatibility. Moreover, hemostatic properties were also evaluated with the use of an in vivo animal model. Swelling ratio was tested at pH = 7.4 and was dependent on the concentration of the surfactant used in the microparticle formulation procedure. The formulation revealed hemocompatibility below 5% which indicates good hemostatic properties. The results of in vivo studies showed lower blood loss and shortened blood clotting time in the case of animals treated with the analyzed microparticles.

Stavarache et al. [176] described mucoadhesive chitosan particles with κ-carrageenan coating attached by polyelectrolyte complexation as potential carrier for 5-aminosalicylic acid. The aim of the study was to deliver the active ingredient to colon without releasing it in upper parts of the gastrointestinal tract. In the first step, chitosan particles were formed with the use of tripolyphosphate crosslinking. Next, the carrageenan coating was applied and crosslinked with KCl. It was shown that the crosslinking time was important for entrapment efficiency which was higher in the case of shorter crosslinking. The drug release tests indicated that the particles without carrageenan coating were not sufficiently acid-resistant and displayed strong burst release effect at pH = 1.2. Moreover, the drug release in the case of carrageenan-coated particles was also slower at pH = 6.8. The obtained results indicate that carrageenan, similarly to other gums, can be useful in formulations intended for colon targeting.

Carrageenan was also applied in the studies aiming at the development of smart nanoparticles revealing the ability to respond to tchanges in different external factors. Geyik and Işıklan [177] described κ-carrageenan-based nanoparticles sensitive to magnetic field, temperature and pH changes. For this purpose, carrageenan was grafted with dimethylaminoethyl methacrylate (DMA) and the product was attached to Fe_3_O_4_ nanoparticle core with microwave-assisted coprecipitation technique. As an active ingredient, 5-fluorouracil was applied. The drug release studies performed at different pH values indicate that in acidic conditions the process was faster and higher amount of active ingredient was released. It was also confirmed that more drug was released in the presence of magnetic field and at higher temperature.

### 4.8. Other Gums

Apart from the gums most extensively investigated in pharmaceutical technology, there are also studies focusing on less popular compounds usually known from other scientific and industrial areas. The other natural polysaccharides are rarely used in unmodified forms or without any additions. In order to obtain micro- or nanogels usually either chemical modifications or the addition of some other polymer is required. Here, we present some examples of the investigation regarding micro- and nanoparticulate systems obtained with locust bean gum, karaya and pullulan.

Kaity and Ghosh [178] prepared microspheres from acrylamide-grafted locust bean gum (LBG) and poly(vinyl alcohol) (PVA). Both polymers formed interpenetrating network. The particles were prepared by emulsion crosslinking with glutaraldehyde. Locust bean gum is a galactomannan and displays relatively high hydrophobicity. The grafting procedure was applied to enhance the hydrophilic character of the compound and increase its miscibility with PVA. The aim of the study was to develop novel controlled-release solid oral dosage form with buflomedil hydrochloride. According to DSC and XRPD experiments results, the drug was dispersed in the polymer matrix molecularly. It was found that the addition of hydrophilic PVA improved the drug encapsulation efficiency, which can be related to polar character of buflomedil hydrochloride. In swelling studies performed at pH values of 1.2 and 6.8 it was shown that the impact of pH was moderate, while the most important factor in swelling behavior of the obtained systems was crosslinking density. The systems with denser crosslink network had weaker ability to swell. The same tendency was observed for the systems with higher amounts of PVA, while hydrophilic drug presence increased swelling rate. Similar trends, regarding the crosslinking density and the amount of PVA, were observed in drug release experiments. In another study [179], the same authors performed stability and toxicity tests for previously investigated particulate systems. They were also subjected to in vivo studies using an animal model. The studies provided no evidence of toxicity and it was confirmed that the active ingredient is released in a sustained way.

A similar system based on pullulan and PVA was tested by Soni and Ghosh [180]. Microparticles were obtained via emulsion crosslinking with glutaraldehyde. Pirfenidon, an active agent approved for the treatment of idiopathic pulmonary fibrosis, was applied as a model drug revealing rapid absorption and rapid elimination from the organism. Similarly to the previously presented studies, the drug release process was strongly dependent on the amount of the crosslinking agent. The increased crosslinking density resulted in a slower drug release process. The same tendency was observed in swelling studies.

An interesting study on pullulan-based nanoparticles applied in nose-to-brain drug delivery was presented by Kashif et al. [181]. Eletriptan-loaded nanoparticles were obtained with coacervation method and crosslinked with ethylene glycol dimethacrylic acid (EGDMA). Similarly to the other gum-based systems, the drug release from pullulan nanoparticles was related to swelling-based diffusion and matrix erosion. At pH = 6.4 burst effect at the initial stage of the study was observed both in the case of nanoparticle suspension and nanoparticle-loaded gel. It was found that the drug release was slower in the case of gel formulation which was related to different properties of a gel medium. However, in vivo tests revealed higher concentrations of the drug in the brain tissue after gel administration which was a result of its prolonged residence time in nasal cavity.

Another formulation containing carboxymethyl LBG was developed by Laha et al. [182]. The particles contained interpenetrating polymer networks of LBG derivative and karaya gum crosslinked with aluminum cations. The formulation was loaded with carvedilol, an antihypertensive agent. The drug release process was studied at pH = 1.2 for the first 2 h and during the next 8 h pH = 6.8 buffer was applied. The formulation displayed initial burst release in acidic conditions followed by sustained release in phosphate buffer. Swelling behavior analysis revealed that the process was slower in an acidic medium which was most probably related to acidic properties of the polymers. At higher pH values the carboxylic groups present in gum molecules become ionized and repulsive forces between negatively charged moieties enhance swelling process. It was also found that matrix properties are strongly correlated with the amount of crosslinking agent. The insufficient amount of cations led to matrix erosion effects and to the release of higher amounts of the drug. The release process followed non-Fickian diffusion model, which is affected by combined simple drug release and polymer relaxation effects. The in vivo studies showed prolonged therapeutic effect of the investigated formulation compared to simple drug suspension. It is noteworthy that the authors analyzed also pH-dependent mucoadhesive properties of the designed formulation. The results indicated that the particles displayed stronger adhesion to mucous membranes in simulated intestinal fluid which could contribute to the observed therapeutic effects.

Shi et al. [183] investigated particles obtained from polyelectrolyte complex of carboxymethyl konjac glucomannan and chitosan derivative. The particle diameter ranged from 600 to 1460 nm, depending on the ratio of both components. The particles were loaded with ovalbumin as a model active ingredient to assess the applicability of the obtained system in oral vaccine formulations. It was found that ovalbumine release rate depended mostly on the presence of konjac gum derivative. However, the study was performed at pH = 7.4 only, so no pH-related effects could be analyzed.

The particular polysaccharide gum applications mentioned in this review are summarized in Table 1.

## 5. Conclusions and Future Directions

Natural gums and their derivatives are a huge and diverse group of compounds with enormous potential regarding drug delivery and other areas. The presented studies indicate that the wide range of possibilities related to the modification of the properties of original compounds is extensively investigated in many studies. Many examples presented in this review describe chemically modified gums, with carboxymethylation among the most popular tool to obtain a compound with altered properties. The introduction of carboxymethyl group allows for the transformation from non-ionic to anionic compound. As the introduced groups are polar, the procedure also leads to increased solubility in water. Moreover, carboxymethylated compounds can also be further modified by polyelectrolyte complexation with some other polymers with opposite electrical charge. Other modifications of physicochemical properties usually include the application of other compounds. The studies summarized here include polymer blends, forming interpenetrating polymer networks (IPNs) or polyelectrolyte complexes. As gums frequently represent anionic properties or can be easily modified to obtain such properties, positively charged chitosan is described as another component of the complex in numerous studies. On the other hand, crosslinking procedures are also popularly applied to obtain gum-based micro- and nanogels. It is important to notice that the studies summarized in this review provide no evidence on toxicity of chemically crosslinked materials, even though glutaraldehyde is considered as relatively toxic. On the other hand, some of the studies dealing with newly synthesized materials do not investigate this issue. It must be emphasized that the studies regarding potential safety concerns are crucial for further application of new substance as a pharmaceutical excipient. In the case of ionotropically crosslinked gums, the toxicity concerns should be raised whenever the selection of crosslinking agent is discussed. Most of the studies deal with salts acceptable in terms of safety; however, in some cases, barium chloride is used as a crosslinker. Even though the investigations regarding these systems are scientifically sound and provide conclusive results, they are of little practical value. As far as the physical crosslinking process is concerned, it is obvious that most of the presented techniques are relatively easy to perform and control at the small laboratory scale. However, the obtained products are non-covalent assemblies and can be susceptible to pH and temperature variations. The removal of crosslinking cations may lead to matrix erosion and the release of the drug. The susceptibility of the dosage form to the alterations of external conditions can be used as an advantage, whenever smart drug delivery systems are designed, but can also exert a negative effect on polymer matrix stability and performance. Moreover, the described physical crosslinking procedures were applied only in small scale. In the case of ionotropic gelation involving dropwise instillation of gum solution in a crosslinking medium, the scale up from laboratory to industrial process could be difficult.

Taking into consideration the potential application of gum-based micro- and nanosystems, it is obvious that they can be administered via different routes. However, the oral route remains the most popular one, which is also reflected in the studies summarized here. Natural gums and their derivatives are usually used as release-modifying agents, as they swell upon contact with water, limiting the drug release rate. For this purpose, the most convenient forms are dried matrices, reconstituting their original micro- and nanogel forms in gastrointestinal tract. In the formulations designed to be administered orally and to release the active ingredient in gastrointestinal tract, the most important issue is the drug release and the answers to the questions regarding the rate and mechanism of the process, as well as the exact drug release region. The investigated compounds are usually pH sensitive, which, considering the physiological pH differences between different parts of gastrointestinal tract, allows for obtaining the formulations releasing the active ingredient at a specific site.

The results of the studies summarized in this review indicate that the drug release process is usually strongly correlated with swelling process. However, some of the analyses focusing on pharmaceutical and medical aspects neglect this important part of physicochemical studies, even though it can provide a lot of useful information regarding drug release. On the other hand, a lot of studies focusing only on physicochemical aspects of the analyzed formulations do not describe in vivo studies which are of crucial importance in terms of future applications. It must be emphasized that the conditions in gastrointestinal tract are unique. In the case of gum-based systems delivered orally, among the factors affecting the drug release rate is mechanical erosion of polymer matrix during the passage. On the other hand, some of the analyzed systems display stronger or weaker mucoadhesive properties, which, in turn, may affect the residence time in particular parts of GI tract. These effects are difficult to be studied in vitro only and to obtain clinically relevant results, animal models should be employed.

## Figures and Tables

**Table 1:** The examples of natural gum-based micro- and nanoparticulate systems.

Drug/Application	Polymer	Fabrication Procedure	Formulation Type	The Most Important Outcomes	Reference
-/antimicrobial applications	Gum arabic	Crosslinking with DVS, modification with DETA and TA	Microgels	Good biocompatibility, low cytotoxicity; antimicrobial effect against *E. coli* and *S. aureus*	[66]
Curcumin	Pectin, carboxymethyl cellulose, gum arabic, carrageenan, alginate	Spray drying	Microgels	Sustained drug release in gastric and intestinal conditions	[67]
Curcumin	Chitosan/gum arabic complex	Precipitation/freeze drying	Pickering emulsion stabilized with nanoparticles	Nanoparticle adsorption at water–oil interface; sustained release of curcumin; low drug degradation rate during storage	[53]
-/potential application in drug delivery	Gum arabic aldehyde/gelatin conjugate	Miniemulsion fusion and Schiff’s base reaction	Nanogels	Good hemocompatibility, low cytotoxicity	[68]
Embelin/ulcerative colitis	Guar gum	Water-in-oil emulsion crosslinked with glutaraldehyde	Dry microparticles	The drug is released mostly in simulated intestinal fluid; in vivo: pretreatment with the formulation prevented colitis development; similar efficacy to mesalamine	[82]
Doxorubicin, metformin hydrochloride/colorectal cancer	Guar gum	Water-in-oil emulsion crosslinked with glutaraldehyde	Dry microparticles	The majority of the drugs was released in colon fluid; polymer matrix displayed sensitivity to the colon microbiome; the particles reached colon without physical decomposition	[75]
Curcumin/ulcerative colitis	PVA, guar gum, alginate, chitosan	Ionotropic gelation, polyelectrolyte complexation, freeze drying	Dry microparticles	Low swelling ability and drug release in gastric fluid compared to intestinal and colon fluids; in vivo: sustained curcumin release, reduced inflammation symptoms, particle accumulation in colon tissue	[83]
5-Fluorouracil/potential anticancer treatment	Chitosan, guar gum grafted with PDMAEMA	Water-in-oil emulsion crosslinked with glutaraldehyde	Dry microparticles	More intensive swelling and drug release at acidic pH; higher drug release rate compared to guar gum particles	[84]
Theophylline/asthma	Carboxymethyl guar gum	Water-in-oil emulsion crosslinked with glutaraldehyde	Dry microparticles	Swelling rate depended on the crosslinking density; the highest drug release rate was observed for the formulations with higher polymer content	[85]
Rhodamine B (model compound)	Carboxymethyl guar gum	Ionotropic crosslinking with STMP	Nanoparticles	The majority of the active ingredient was released at pH = 7.4; non-toxicity below the concentration of 0.3 mg/mL	[86]
Ag85A/immunization against tuberculosis	Guar gum	Nanoprecipitation, spray drying	Nanoparticles	The majority of the antigen was released in intestinal environment (in vivo); increased intake of the active from the intestines; increased IgG and IgA levels	[87]
Borneol/central nervous system diseases	Guar gum/vitamin E conjugate	Self-assembly	Nanoparticle	Good biocompatibility and hemocompatibility of placebo nanoparticles; the results were worse for a drug-loaded system	[88]
Mesalamine/ulcerative colitis	Alginate, HPMC, Eudragit^®^ FS-30D	Water-in-oil emulsion crosslinked with CaCl_2_, particles loaded to Eudragit^®^-coated HPMC capsule	Microparticles loaded to enteric-coated capsule	Burst drug release in acidic pH in the case of microparticles alone; increased drug release in simulated intestinal fluid for capsule	[98]
5-Fluorouracil/colorectal cancer	Alginate, chitosan, κ-carrageenan	Ionotropic alginate gelation with CaCl_2_, coating by polyelectrolyte complexation	Dry microparticles	Swelling in acidic pH depended on the coating type; swelling and drug release was faster in intestinal and colon media for all formulations	[99]
Doxorubicin/cancer	Alginate	Ionotropic gelation with CaCl_2_ in microfluidic device	Microgels	The particle size can be controlled with the device parameters; the formulation displayed good efficacy towards breast cancer cells	[100]
Insulin/diabetes	Acetalated dextran, alginate	Ionotropic gelation with CaCl_2_ (electro-spray system)	Microgels loaded with nanoparticles	The amount of released insulin depended on glucose level in surrounding medium; alginate protected insulin from degradation; in vivo: sufficient clinical efficacy during 22 days of study	[104]
-/model microgels	Gellan, chitosan	Ionotropic gelation with CaCl_2_ or KCl, coating with chitosan	Microgels	Good stability in aqueous media, except for KCl-crosslinked microgels; the particles were stable in gastric conditions; chitosan-coated microgels were less susceptible to degradation in intestinal fluid	[110]
Prednisolone, paclitaxel/cancer	Gellan gum/prednisolone conjugate	Self-assembly	Nanogels	Prednisolone acted as a hydrophobic moiety in nanogel self-assembly; increased cytotoxic efficacy towards different cancer cell lines	[111]
Curcumin/cancer	Gellan gum, chitosan	Polyelectrolyte complexation	Nanogels	Prolonged curcumin release; good hemocompatibility and non-toxicity	[112]
Piroxicam/non-melanoma skin cancers	Gellan gum/cholesterol/riboflavin conjugate	Self-assembly	Nanogels	Nanogels enhanced the drug retention in epidermis; nanogels permeated across stratum corneum and release the drug in viable epidermis	[113]
Probiotic bacteria/gut microbiota disbiosis	Gellan gum, xanthan gum	Ionic crosslinking with CaCl_2_, freeze drying	Microcapsules	Improved survival rate during simulated gastrointestinal tract passage	[114]
Capecitabine/colorectal cancer	Xanthan gum	Crosslinking with glutaraldehyde	Dry microparticles	Floating microparticles with mucoadhesive properties were obtained; sustained release in simulated gastric fluid; significant drug bioavailability increase	[121]
Bovine serum albumin/model for protein delivery	Carboxymethyl xanthan gum	Ionotropic crosslinking with AlCl_3_	Microparticles	Additional coating was necessary to reduce protein release in simulated gastric fluid; the protein stability was unaffected by encapsulation	[123,124]
Diltiazem/model drug: angina pectoris, hypertension, arrythmia	Carboxymethyl xanthan gum	Ionotropic crosslinking with AlCl_3_ or glutaraldehyde	Microparticles	The treatment with polyethylene imine reduced drug release in simulated gastric fluid; slowest drug release for sequential crosslinking with AlCl_3_ and glutaraldehyde	[125,126,127]
Glibenclamide/diabetes	Carboxymethyl xanthan gum, alkyl chain-grafted-xanthan copolymer	Ionotropic crosslinking with AlCl_3_	Microparticles loaded with copolymer micelles	Sustained drug release and prolonged blood glucose reduction effect; no in vivo difference between free and micellar drug	[128]
Repaglinide/diabetes	Carboxymethyl xanthan gum, carboxyethyl xanthan gum	Ionotropic crosslinking with AlCl_3_	Dry microparticles	Carboxyethyl derivative alone was unsuitable for hydrogel production; additional hydrophobization of hydrogel surface via acetylation was necessary to slow down the release; stable glucose lowering effect over 8 h	[122]
Theophylline/model drug	Glicydyl methacrylate grafted xanthan gum	Covalent crosslinking with dimethacrylates	Microparticles	Side chain length affected the particle surface; grafted xanthan alone displayed faster drug release ability	[129]
Theophylline/model drug	Xanthan gum, gelatin	Water-in-oil emulsion crosslinking with glutaraldehyde	Dry floating microspheres	Higher xanthan gum content resulted in better buoyancy and improved encapsulation efficiency	[144]
Doxorubicin/cancer	Cystamine tetra-acylhydrazine—grafted xanthan gum	Covalent crosslinking with cystamine tetra-hydrazide	Nanogels	pH- and redox-responsive drug release in medium simulating cancer cells environment; nanoparticles uptake by endocytosis	[130]
Doxorubcin/cancer	2-Acrylamido-2-methylpropane sulfonic acid grafted xanthan gum	Covalent crosslinking with *N*,*N*-bis(acryolyl) cystamine	Nanoparticles	The drug release could be controlled with redox and pH	[131]
Ketoprofen/musculoskeletal and joint disorders	Polyacrylamide-grafted-xanthan gum	Ionotropic crosslinking with AlCl_3_	Dry microparticles	pH-dependent extended drug release at pH = 6.8; the efficacy of the formulation and safety towards gastric mucosa was confirmed	[132]
Curcumin/inflammatory bowel diseases	Polyacrylamide-grafted-xanthan gum	Ionotropic crosslinking with AlCl_3_	Dry nanoparticles	The drug release was faster in simulated colon environment; in vivo: the formulation performed similarly or better than standard sulfasalazine	[134]
Mesalamine/ulcerative colitis	Xanthan gum, guar gum	Oil-in-water emulsion crosslinked with glutaraldehyde	Microparticles	The drug release rate depended on microbiota composition and condition	[135]
Celecoxib/various inflammatory conditions, colorectal cancer chemoprevention	Xanthan gum, locust bean gum	Oil-in-water emulsion crosslinked with glutaraldehyde	Dry microparticles	Extended release was comparable to commercial tablets; locust bean gum decreased the drug release rate	[136]
Metoprolol/myocardial infarction, heart failure, hypertension	Xanthan gum, locust bean gum, pectin	Ionotropic gelation with CaCl_2_	Dry microparticles	Confirmed in vitro buoyancy and gastroretention in vivo; pulsatile drug delivery with lag phase at pH = 1.2 and burst release at 6.8	[137]
*Calendula officinalis* extract/cosmetic applications	Gellan gum, xanthan gum	Ionotropic gelation with CaCl_2_ (extrusion or emulsion)	Microspheres	Size and entrapment efficiency of microspheres depended on the fabrication method	[138]
Diclofenac/model drug	Carboxymethyl xanthan gum, carboxymethyl cellulose	Ionotropic gelation with different salts	Microparticles	Carboxymethyl cellulose reduced entrapment efficiency; the drug release rate depended on the crosslinking agent concentration and time	[139]
Diclofenac/model drug	Xanthan gum, polyvinyl alcohol	Oil-in-water emulsion crosslinked with glutaraldehyde	Microparticles	Prolonged release of the drug; the amounts of crosslinking agent and xanthan gum affected the entrapment efficiency and drug release; good correlation between in vitro and in vivo data	[143]
*Thymus vulgaris*, *Rosmarinus officinalis* and *Syzygium aromaticum* essential oils/antimicrobial effect	Chitosan + xanthan gum/guar gum/alginate/pectin	Oil-in-water emulsion crosslinked with tripolyphosphate	Nanocapsules	Burst release in 3 h followed by extended phase; decreased antibacterial activity compared to pure essential oils	[141]
Cinnamon essential oil/antimicrobial effect	Chitosan, xanthan gum	Polyelectrolyte complexation, crosslinking with glutaraldehyde	Microcapsules	Improved thermal stability of essential oil; activity against *S. aureus* and *E. coli*	[142]
Rivastigmine/Alzheimer’s disease	Tragacanth	Ionotropic gelation with CaCl_2_ or BaCl_2_	Dry microparticles	pH-dependent drug release and swelling behavior; higher amounts of the drug released in simulated intestinal conditions	[156]
Diclofenac sodium/model drug	Carboxymethyl tragacanth	Ionotropic gelation with AlCl_3_	Microparticles	pH-dependent swelling behavior and drug release; the highest swelling degree at pH = 7.0; minimal cytotoxicity	[157]
Cisplatin/cancer	Poly(itaconic acid)-grafted tragacanth	water-in-oil nanoemulsion antisolvent precipitation	Lecithin-coated nanoparticles	Different amounts of cisplatin were released at pH = 2.5 and 6.5 due to the differences in swelling behavior; non-toxic character of the carrier; good cytotoxicity towards cancer cell lines	[158]
Methotrexate/cancer	De-esterified tragacanth, chitosan	Polyelectrolyte complexation (complex coacervation)	Nanoparticles	The drug was released in a sustained manner; worse cytotoxicity results compared to non-encapsulated drug; selective nanoparticles uptake by human breast cancer line cells	[159]
Bovine serum albumin/model protein	κ-Carrageenan, alginate	Ionotropic gelation with CaCl_2_ and KCl	Microparticles	pH-dependent drug release pattern; only 10% of the active ingredient was release in acidic conditions	[174]
Tannic acid/hemorrhage	κ-Carrageenan, gelatin	Polyelectrolyte complexation	Dry microparticles	High swelling in physiological pH, good biocompatibility and hemostatic properties confirmed by the results of in vivo tests	[175]
5-Aminosalicylic acid/inflammatory bowel disease	κ-Carrageenan, chitosan	Ionotropic gelation, polyelectrolyte complexation	Dry microparticles	Carrageenan coating prevented burst drug release at acidic pH and decreased the amount of the drug released in simulated intestinal fluid	[176]
5-Fluorouracil/cancer	κ-Carrageenan grafted with poly(dimethylaminoethyl methacrylate)	Microwave-assisted co-precipitation	Nanoparticles	Higher amounts of the drug were released at acidic pH, in the presence of magnetic field and at higher temperature	[177]
Buflomedil hydrochloride/model drug	Acrylamide-grafted locust bean gum, PVA	Emulsion crosslinking with glutaraldehyde	Microparticles	PVA improved drug entrapment efficiency; the impact of pH on the swelling behavior was moderate; crosslinking density was the most important factor determining the drug release rate; formulation was non-toxic and biocompatible	[178,179]
Pirfenidon/idiopathic pulmonary fibrosis	Pullulan gum, PVA	Emulsion crosslinking with glutaraldehyde	Microparticles	The drug release rate depended mostly on the crosslinking density	[180]
Eletriptan/migraine (nose-to-brain formulation)	Pullulan gum crosslinked with EGDMA	Covalent crosslinking	Microparticles	The drug release rate was correlated with matrix swelling and erosion; burst drug release at pH = 6.4; slower drug diffusion and higher residence time in nasal cavity observed for gel	[181]
Carvedilol/hypertension	Locust bean gum, karaya gum	Ionotropic gelation with aluminum cations	Microparticles	Initial burst release in acidic pH followed with sustained release at pH = 6.8; higher drug release in the case of lower crosslinking density; stronger adhesion to intestinal mucous membrane; in vivo efficacy comparable to plain drug suspension	[182]
Ovalbumin/model compound	Carboxymethyl konjac glucomannan, chitosan derivative	Polyelectrolyte complexation	Microparticles	Albumin release depended mostly on konjac gum derivative content	[183]

## Data Availability

Not applicable.
